# Activation of methionine metabolism mediated by HNF4α confers ferroptosis resistance in hepatocellular carcinoma

**DOI:** 10.1038/s41420-026-03165-0

**Published:** 2026-05-26

**Authors:** Xiaolei Zhou, Zhenzhen Li, Libo Wang, Yongdeng Xu, Pinsheng Han, TianYu Zhao, Sen Liu, Liuyang Zhu, Miao Zhang, Chenglong Chu, Xiulin Yi, Fengying Yan, Tao Cui, Xiaoliang Wang, Ze Wang

**Affiliations:** 1 State Key Laboratory of Druggability Evatuation and Systematic Translational Medicine, Tianjin Institute of Pharmaceutical Research, Tianjin, 300301 China; 2 First Central Clinical College of Tianjin Medical University, Tianjin, 300192 China; 3 Nankai University of Medicine College, Tianjin, 300192 China; 4 Department of Pharmacology, Shenyang Pharmaceutical University, Shenyang, 110016 China; 5 Tianjin Joint Innovation Biotechnology Co. Ltd., Tianjin, 300301 China; 6 Tianjin Third Central Hospital, Tianjin, 300170 China; 7 Hefei Tianhui Biotechnology Co. Ltd., Hefei, 230000 China

**Keywords:** Apoptosis, Transporters

## Abstract

Hepatocellular carcinoma (HCC) remains a leading cause of cancer-related mortality worldwide. Ferroptosis induction represents a promising therapeutic strategy, but its efficacy is constrained by tumor heterogeneity, particularly the relative resistance of epithelial cancer cells to ferroptosis inducers. In this study, we explore the mechanisms of epithelial HCC cells (EC) acquire resistance to ferroptosis. Transcriptomics and metabolomics analyses revealed that the cysteine and methionine metabolic pathway is associated with the resistance of EC to ferroptosis inducer imidazole ketone erastin (IKE). Cystine and methionine limitation experiments confirmed that the sensitivity of EC to IKE is related to methionine metabolism. Inhibiting the activity or expression of major methionine metabolic enzymes markedly enhanced the cell death and oxidative stress levels of EC. The analysis results of multiple public datasets indicate that the expression of the major methionine metabolic enzymes is related to hepatocyte nuclear factor 4α (HNF4α). Chromatin immunoprecipitation (ChIP) and luciferase assays were employed to confirm the transcriptional regulatory role of HNF4α on the major methionine metabolic enzymes. Additionally, in vitro RNA interference experiments and in vivo xenograft mouse models verified that down-regulation of HNF4α abolished ferroptosis resistance in EC. In conclusion, HNF4α-mediated activation of methionine metabolism sustains cellular antioxidant capacity, thereby promoting ferroptosis resistance in epithelial HCC cells. These findings position HNF4α and major methionine metabolic enzymes as potential therapeutic targets to overcome ferroptosis resistance in HCC, offering a rational basis for more effective treatment strategies.

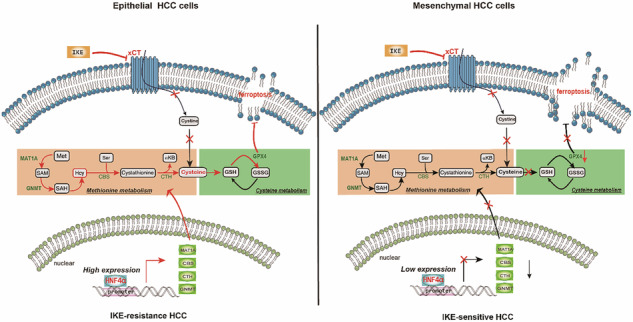

## Introduction

It is widely recognized that hepatocellular carcinoma (HCC) is the most prevalent intra-abdominal malignancy, ranking fifth in incidence and third in mortality worldwide [1]. At present, the incidence of HCC in China accounts for approximately 55% of cases worldwide, posing a significant threat to the life and health of the Chinese population [2].

Ferroptosis is a distinctive form of oxidative cell death generated by the iron-dependent accumulation of lipid reactive oxygen species [3, 4]. Induction of ferroptosis in cancer cells has emerged as a promising therapeutic strategy for cancer treatment [5, 6]. The xCT-GSH-GPX4 axis represents the most important antioxidant pathway by which cells avoid ferroptosis [7]. Cells transport cystine through SLC7A11 (also referred to as xCT), which plays an essential role in glutathione synthesis (GSH) biosynthesis and consequently enhances the cellular antioxidant defense mechanism [8, 9]. Glutathione peroxidase (GPX4) reduces lipid hydroperoxides to lipid alcohols using GSH, thereby inhibiting tumors ferroptosis [3]. Imidazole ketone erastin (IKE), a classical inhibitor of xCT, exerts anti-tumor effects as a ferroptosis inducer [10]. In addition, recent studies have shown that although cancer cells mainly rely on xCT to uptake cystine from the microenvironment to replenish intracellular GSH [9], some cancer cells can also increase intracellular GSH levels through methionine metabolism [11–13]. Methionine (Met) plays a crucial role in maintaining metabolic balance, supporting growth and development, and modulating the immune system [14]. Methionine adenosyltransferase 1 A (MAT1A) converts Met to S-adenosylmethionine (SAM). S-adenosylhomocysteine (SAH) is subsequently generated from SAM by glycine N-methyltransferase (GNMT). SAH then undergoes hydrolysis to form homocysteine (Hcy). Homocysteine is converted to cystathionine (Ctt) by cystathionine β-synthase (CBS), and subsequently to cysteine (Cys) by cystathionine γ-lyase (CTH) [15, 16]. Cysteine further contributes to the production of the antioxidant GSH during cysteine metabolism [16–18].

However, during the dynamic progression of cancer, tumors often develop heterogeneity and resistance to therapies [19, 20]. Due to this heterogeneity, tumors may contain a variety of cell populations with distinct molecular signatures and different sensitivities to treatment, which may restrict the efficacy of ferroptosis-based therapies [21, 22]. Numerous studies have shown that epithelial cancer cells exhibit greater resistance to ferroptosis than mesenchymal cancer cells [23–25]. However, the mechanisms underlying ferroptosis resistance in epithelial cancer cells remain unclear. In this study, we demonstrated that mesenchymal HCC cells (MC) are more sensitive to IKE than epithelial HCC cells (EC). Hepatocyte nuclear factor 4α (HNF4α) is a member of the nuclear receptor transcription factor family and serves as a critical regulator of liver-specific gene expression. It plays an essential role in maintaining hepatocyte identity and ensuring proper liver function [18, 26]. This study establishes a link between HNF4α and methionine metabolism in HCC [18]. High expression of HNF4α in EC may supplement intracellular GSH through a non-xCT-dependent pathway by activating metabolic enzymes involved in methionine metabolism, which may represent a key mechanism underlying ferroptosis resistance in EC.

The present study reveals a significant contributor to ferroptosis resistance in EC and provides a strong foundation for future investigations into clinical translation, combination therapies, and the role of tumor heterogeneity.

## Results

### Ferroptosis resistance of EC is associated with methionine metabolism

**Fig. 1:**
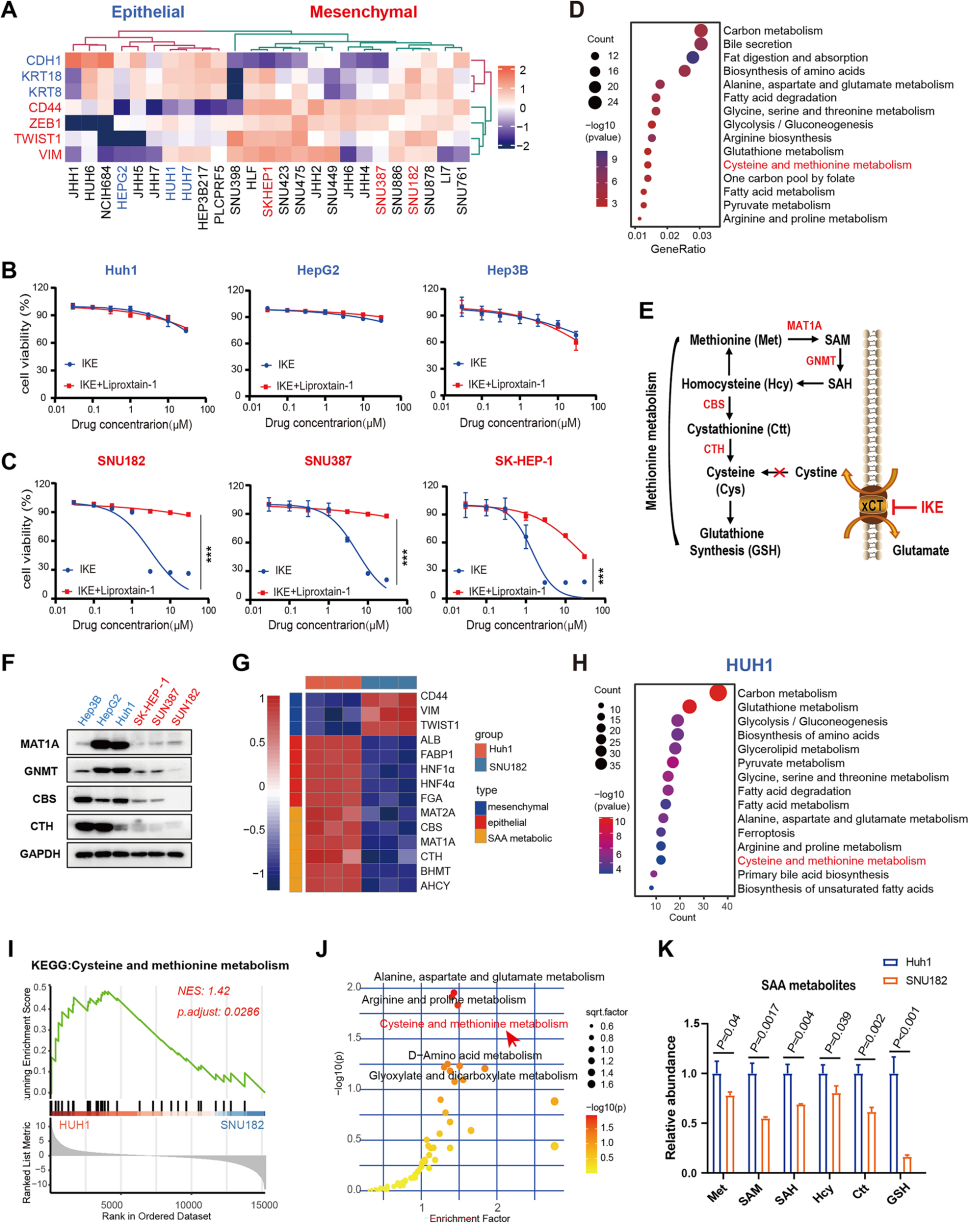
Major methionine metabolic enzymes are highly expressed in EC. **A** Hepatocellular carcinoma cells were clustered according to EMT markers. The mRNA levels of epithelial markers (blue) and mesenchymal markers, including EMT-related transcription factors (red), were visualized using a heatmap generated in R. **B**, **C** Three EC lines (Huh1, Hep3B, and HepG2) and three mesenchymal HCC cell lines (MC) (SK-HEP-1, SNU182, SNU387) were treated with IKE (0.03, 0.1, 0.3, 1, 3, 10, 30 µM) for 24 h, with or without co-treatment with the ferroptosis inhibitor Liproxstatin-1 (2 µM) (n = 3). Relative cell viability was assessed using an MTS assay, ***p < 0.0001. **D** KEGG enrichment analysis of genes highly expressed in epithelial cells from the CCLE liver cancer dataset. **E** Schematic representation of methionine metabolism. **F** Western blot analysis of MAT1A, GNMT, CBS, and CTH expression in EC and MC (n = 3). **G** Heatmap of EMT- and methionine metabolism-related gene expression. **H** KEGG enrichment of highly expressed genes in Huh1 cells. **I** GSEA of differential expression between Huh1 and SNU182 cells using KEGG gene sets. The enrichment score (ES) is indicated by the green line, and black lines denote genes involved in cysteine and methionine metabolism. Normalized enrichment score (NES) and adjusted p-value are shown in the top-right corner. **J** Differential metabolites between SNU182 and Huh1 cells were significantly enriched for methionine metabolism. **K** LC-MS/MS analysis of methionine metabolism-related metabolites in Huh1 and SNU182 cells (n = 3).

To examine the differences between epithelial and mesenchymal HCC cells, we performed cluster analysis of RNA-seq data from 25 human HCC cell lines obtained from the Broad Institute Cancer Cell Line Encyclopedia (CCLE) database. Based on epithelial markers (CDH1, KRT8, and KRT18), mesenchymal markers (CD44 and vimentin), and transcription factors regulating EMT (ZEB1 and TWIST1) [27–29], these HCC cell lines were classified into two distinct groups (10 epithelial vs. 15 mesenchymal) (Fig. 1A).

Our data confirmed that, compared with epithelial hepatocellular carcinoma cells (EC) (HepG2, Huh1, and Hep3B) (Fig. 1B), mesenchymal hepatocellular carcinoma cells (MC) (SK-HEP-1, SNU182, and SNU387) were more sensitive to IKE. However, the ferroptosis inhibitor Liproxstatin-1 counteracted this effect (Fig. 1C). These findings are consistent with previous reports that epithelial cancer cells are more resistant to ferroptosis than mesenchymal cancer cells. Therefore, it is necessary to further investigate the mechanisms underlying ferroptosis resistance in EC.

Kyoto Encyclopedia of Genes and Genomes (KEGG) enrichment analysis demonstrated that highly expressed genes in the 10 EC from the CCLE database were significantly enriched in the cysteine and methionine metabolism pathway (Fig. 1D). Accordingly, we analyzed key metabolic enzymes involved in methionine metabolism, including methionine adenosyltransferase 1 A (MAT1A), glycine N-methyltransferase (GNMT), cystathionine β-synthase (CBS), and cystathionine γ-lyase (CTH), which contribute to cysteine generation (Fig. 1E). The expression levels of these major methionine metabolic enzymes were significantly increased in EC (Fig. S1A). Furthermore, the expression levels of these enzymes were significantly higher in primary HCC tumors than in metastatic tumors. This observation may imply that primary tumors (often more epithelial-like) rely on this pathway, whereas metastatic tumors (which are frequently more mesenchymal-like) do not, which is consistent with the overall hypothesis of this study (Fig. S1B).

The expression levels of the major methionine metabolic enzymes were significantly higher in EC than in MC (Fig. 1F). Cluster analysis of RNA-seq data from Huh1 and SNU182 cells also showed that Huh1 cells exhibited higher expression levels of methionine metabolism-related genes than SNU182 cells (Fig. 1G). In addition, KEGG enrichment analysis demonstrated that Huh1 cells (Fig. 1H), but not SNU182 cells (Fig. S1C), were significantly enriched in cysteine and methionine metabolism. Gene Set Enrichment Analysis (GSEA) yielded similar results (Fig. 1I). Furthermore, metabolomic analysis confirmed that the differential metabolites between Huh1 and SNU182 cells were significantly enriched in cysteine and methionine metabolism (Fig. 1J). LC-MS/MS assays further verified that Huh1 cells exhibited higher intracellular levels of Met, SAM, SAH, Hcy, Ctt, and GSH than SNU182 cells (Fig. 1K). Moreover, compared with normal tissue, the expression of major methionine metabolic enzymes was reduced in HCC tissue (Fig. S1D). As shown in Supplementary Fig. 1E, HCC patients with high expression levels of these major methionine metabolic enzymes exhibited better prognostic outcomes than patients with low expression levels. Taken together, these results indicate that major methionine metabolic enzymes are significantly upregulated in EC and are associated with ferroptosis resistance and patient prognosis.

### Methionine restriction enhances ferroptosis sensitivity in EC

**Fig. 2:**
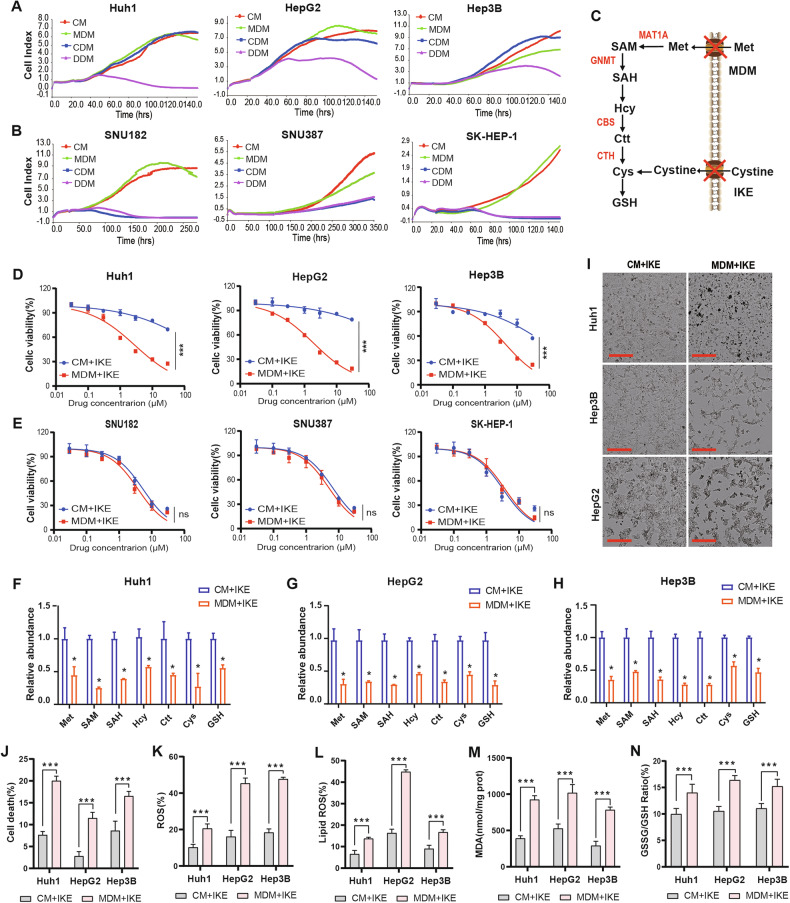
Methionine restriction enhances the sensitivity of EC to IKE. **A**, **B** Methionine and cystine restriction experiments were performed as described in Methods (n = 3). EC (**A**) and MC (**B**) were cultured in complete medium (CM, red), methionine-deficient medium (MDM, green), cystine-deficient medium (CDM, blue), or double-deficient medium (DDM, purple). Cell proliferation was monitored in real time using the RTCA eSight system (Agilent) (n = 3). **C** Schematic of methionine and cystine restriction experiments. Cell viability of EC (**D**) and MC (**E**) was assessed after 24 h co-culture with IKE (0.03, 0.1, 0.3, 1, 3, 10, 30 µM) in CM (blue) or MDM (red), measured by MTS assay (n = 3). **F**-**H** EC were cultured with 10 µM IKE in CM (blue) or MDM (orange) for 24 h, and LC-MS/MS was used to quantify metabolites involved in methionine metabolism (n = 3), *p < 0.005. Three EC lines were cultured in CM or MDM with 10 µM IKE for 24 h and analyzed by microscopy (**I**), scale bar = 200 μm, and by Propidium Iodide (PI, 2 µM) staining to assess cell death (**J**). Intracellular ROS was measured using H2DCFDA (1 µM) (**K**), lipid ROS using C11-BODIPY (581/591, 2 µM) (**L**), MDA levels as a marker of lipid peroxidation (**M**), and the GSSG/GSH ratio (**N**) was used to assess cellular redox status (n = 3). Fluorescence intensities of PI, H2DCFDA, and C11-BODIPY were measured by Cytek NL-CLC full-spectrum flow cytometry, ***p < 0.001.

The uptake of methionine and cystine was blocked using methionine-deficient medium (MDM) and cystine-deficient medium (CDM), respectively. The results showed that depletion of both methionine and cystine led to cell proliferation arrest or cell death in EC and MC (Fig. 2A, B). Nevertheless, the absence of methionine or cystine alone did not affect the proliferation of EC. The absence of methionine also did not affect the proliferation of MC, whereas the absence of cystine significantly inhibited the proliferation of MC. These findings suggest that EC may possess additional regulatory mechanisms that compensate for cystine limitation.

As shown in Fig. 2C, three types of EC were treated simultaneously with MDM and IKE. Inhibition of methionine metabolism through methionine restriction increased sensitivity to IKE in EC but not in MC (Fig. 2D, E). LC-MS/MS analysis confirmed that the levels of Met, SAM, SAH, Hcy, Ctt, Cys, and GSH were significantly decreased in EC following methionine restriction and IKE treatment (Fig. 2F–H). Methionine restriction also significantly enhanced the cytotoxic effect of IKE in Huh1, HepG2, and Hep3B cells (Fig. 2I, J), and increased intracellular total reactive oxygen species (ROS) (Fig. 2K), lipid ROS (Fig. 2L), and malondialdehyde (MDA) levels (Fig. 2M), which are routine biomarkers for the detection of ferroptosis [30]. In addition, the ratio of oxidised glutathione (GSSG) to reduced glutathione (GSH) serves as a crucial indicator for assessing the cellular redox state [31]. After methionine restriction and IKE treatment, the GSSG/GSH ratio in the three types of EC increased significantly (Fig. 2N). These results indicate that methionine metabolism plays an important role in EC. EC can compensate for cystine restriction through methionine metabolism to resist ferroptosis and maintain cell viability, whereas this mechanism is absent in MC. Importantly, ferroptosis sensitivity in EC was significantly increased following methionine restriction.

### Inhibition of methionine metabolic enzymes sensitizes EC to ferroptosis

**Fig. 3:**
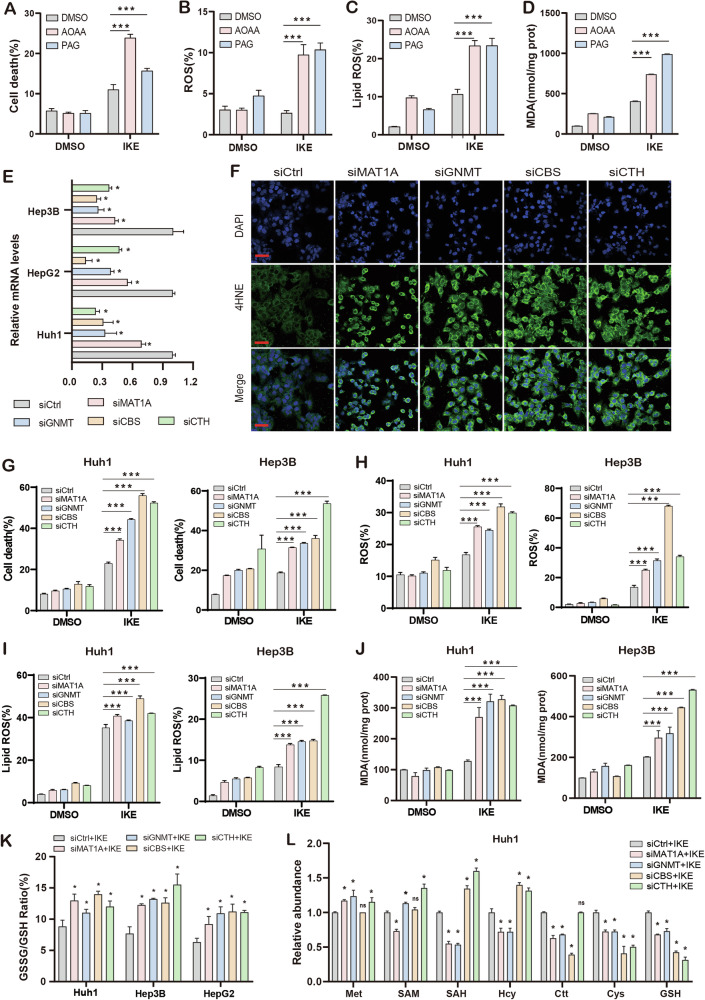
Inhibition of methionine metabolism enhances the efficacy of ferroptosis inducers. **A**–**D** Huh1 cells were treated with IKE (10 µM) or DMSO, in combination with CBS or CTH inhibitors (AOAA, PAG, 5 µM) for 24 h. Cell death was assessed by Propidium Iodide (PI, 2 µM) staining (**A**), total ROS levels by H2DCFDA (1 µM) (**B**), lipid ROS by C11-BODIPY (581/591, 2 µM) (**C**), and MDA, a lipid peroxidation marker, was quantified (**D**) (n = 3), ***p < 0.0001. (E) After 24 h of siRNA transfection, MAT1A, GNMT, CBS, and CTH expression in EC was measured by RT-qPCR (n = 3), *p < 0.0001. **F** Huh1 cells were transfected with siRNA for 24 h, treated with IKE (10 µM) for 10 h, and intracellular 4-Hydroxynonenal (4HNE, green) was detected by immunofluorescence. Nuclei were stained with DAPI (blue); scale bar = 50 µm. Following 24 h siRNA transfection, Huh1 and Hep3B cells were incubated with IKE (10 µM) or DMSO for 24 h, and cell death (**G**), total ROS (**H**), lipid ROS (**I**), and MDA levels (**J**) were assessed (n = 3). Fluorescence intensities were measured by Cytek NL-CLC full-spectrum flow cytometry, ***p < 0.001. **K** GSSG/GSH ratio in Huh1, Hep3B, and HepG2 cells was measured after siRNA transfection and 24 h IKE treatment (n = 3). **L** LC-MS/MS analysis of methionine metabolism-related metabolites in Huh1 cells after siRNA transfection and IKE treatment (n = 3), ns indicates no significant difference, *p < 0.001.

Inhibitors of CBS or CTH (AOAA and PAG) significantly enhanced the cytotoxic effect of IKE in Huh1 cells (Fig. 3A), as well as in HepG2 and Hep3B cells (Fig. S2A). These treatments also increased intracellular total ROS (Fig. 3B, Fig. S2B), lipid ROS (Fig. 3C, Fig. S2C), and MDA levels (Fig. 3D, Fig. S2D). The expression levels of major methionine metabolic enzymes (MAT1A, GNMT, CBS, and CTH) in three types of EC were knocked down by RNA interference, and the knockdown efficiency was verified by RT-qPCR (Fig. 3E). Compared with siCtrl EC, EC with knockdown of MAT1A, GNMT, CBS, and CTH exhibited increased levels of 4-Hydroxynonenal (4HNE) (Fig. 3F, Fig. S2E-F). Similar to MDA, intracellular 4HNE levels are positively correlated with the degree of oxidative damage and can therefore indirectly reflect ferroptosis. Meanwhile, downregulation of the major methionine metabolic enzymes in EC enhanced the cytotoxic effect of IKE (Fig. 3G, Fig. S2G). It also increased intracellular total ROS (Fig. 3H, Fig. S2H), lipid peroxidation (Fig. 3I, Fig. S2I), and MDA levels (Fig. 3J, Fig. S2J). When the expression of major methionine metabolic enzymes was downregulated in the three types of EC followed by IKE treatment, the GSSG/GSH ratio increased significantly (Fig. 3K). Meanwhile, the levels of Met increased, whereas those of Cys and GSH decreased (Fig. 3L, Fig. S2K–L). In addition, staining of dead cell nuclei using the eTox Red reagent further confirmed that downregulation of major methionine metabolic enzymes significantly enhanced IKE-induced ferroptosis (Fig. S3A–C). In summary, inhibition of the major methionine metabolic enzymes increased the sensitivity of EC to ferroptosis inducers.

### The expression of major methionine metabolic enzymes is regulated by HNF4α

**Fig. 4:**
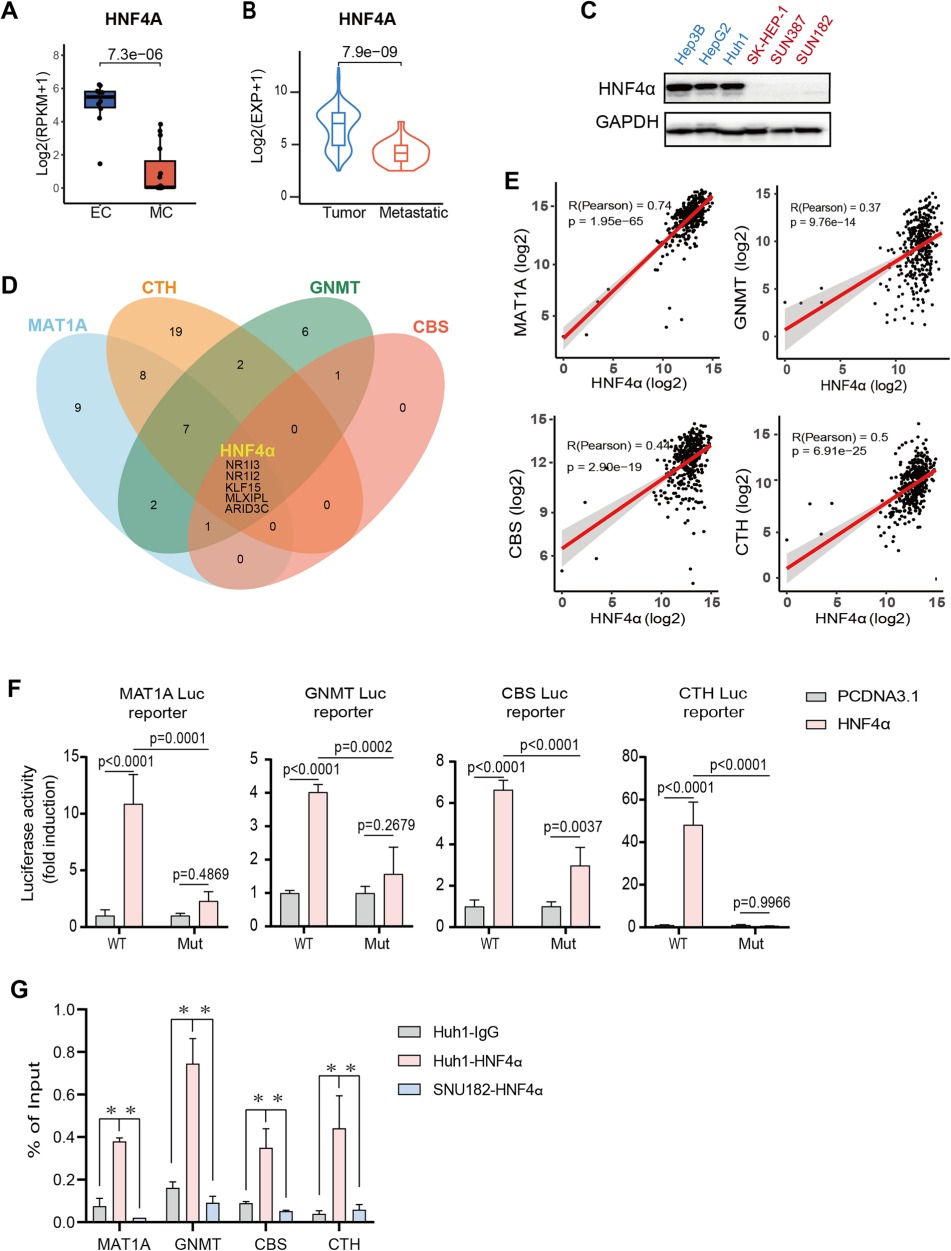
HNF4α directly binds to and transactivates the promoters of major methionine metabolism enzymes. **A** HNF4α expression in EC and MC from the CCLE liver cancer dataset. **B** HNF4α expression in primary tumor and metastatic liver cancer tissues, obtained from TNMplot and visualised using R package ggplot2. **C** Western blot analysis of HNF4α expression in EC (HepG2, Huh1, Hep3B) and MC (SK-HEP-1, SNU182, SNU387). **D** Intersection of transcription factors significantly correlated with major methionine metabolism enzymes in TCGA-LIHC dataset; numbers indicate counts of overlapping transcription factors. **E** Scatter plots showing positive correlations between major methionine metabolism enzymes and HNF4α in TCGA-LIHC patients; p values and Pearson correlation coefficients were calculated using R. **F** Luciferase reporter activity in SNU182 cells, showing reduced activity in deletion mutants upon HNF4α overexpression. **G** ChIP-qPCR analysis of HNF4α binding to promoters of major methionine metabolism enzymes in Huh1 and SNU182 cells. Negative controls included Huh1-IgG and SNU182-HNF4α (n = 3), *p < 0.0001.

Transcription factor enrichment analysis and Transcriptional Regulatory Relationships Unraveled by Sentence-based Text mining (TRRUST) analysis showed that the differentially expressed genes between EC and MC were significantly enriched for HNF4α (Fig. S4A-B). HNF4α expression was significantly elevated in EC compared with MC in the CCLE liver cancer dataset (Fig. 4A). In addition, HNF4α expression was higher in patients with primary tumors than in those with metastasis (Fig. 4B). We assessed HNF4α expression levels in EC and MC samples using Western blot analysis, and the results were consistent with the analysis of the CCLE dataset (Fig. 4C). We further analyzed transcription factors involved in regulating the four major methionine metabolic enzymes in The Cancer Genome Atlas (TCGA) Liver Hepatocellular Carcinoma (LIHC) dataset and identified their intersection (Fig. 4D). The major methionine metabolic enzymes showed a significant positive correlation with HNF4α (Fig. 4E). Moreover, the expression levels of mesenchymal markers (CD44 and vimentin) and transcription factors regulating EMT (TWIST1 and SNAI1) were negatively correlated with HNF4α (Fig. S4C-D). Notably, similar to the major methionine metabolic enzymes (Fig. S1E), patients with elevated HNF4α expression levels exhibited improved survival outcomes (Fig. S4E). In addition, compared with normal individuals, the expression level of HNF4α was decreased at different stages in patients with HCC (Fig. S4F). These findings indicate that HNF4α contributes to the regulation of the expression of major methionine metabolic enzymes in HCC.

To further examine this hypothesis, ENCODE analysis of published ChIP-seq datasets demonstrated that the promoters of MAT1A, GNMT, CBS, and CTH are bound by HNF4α (Fig. S5A–D). Wild-type (WT) and deletion mutant (Mut) clones were constructed based on enrichment scores and the localisation of peak sites (Fig. S5E). We confirmed that overexpression of HNF4α in SNU182 cells significantly enhanced the luciferase activity of reporter genes driven by the MAT1A, GNMT, CBS, or CTH promoters containing HNF4α binding sites. However, upon deletion of the binding site, this enhancement was markedly attenuated (Fig. 4F). In addition, ChIP-qPCR analysis confirmed that HNF4α bound to the promoters of the major methionine metabolic enzymes in EC but not in SNU182 cells (Fig. 4G, Fig. S5F-G). Taken together, HNF4α plays an important role in regulating the expression of major methionine metabolic enzymes and may serve as a critical transcription factor underlying the differential ferroptosis sensitivity between EC and MC.

### Inhibition of HNF4α induces ferroptosis of EC

**Fig. 5:**
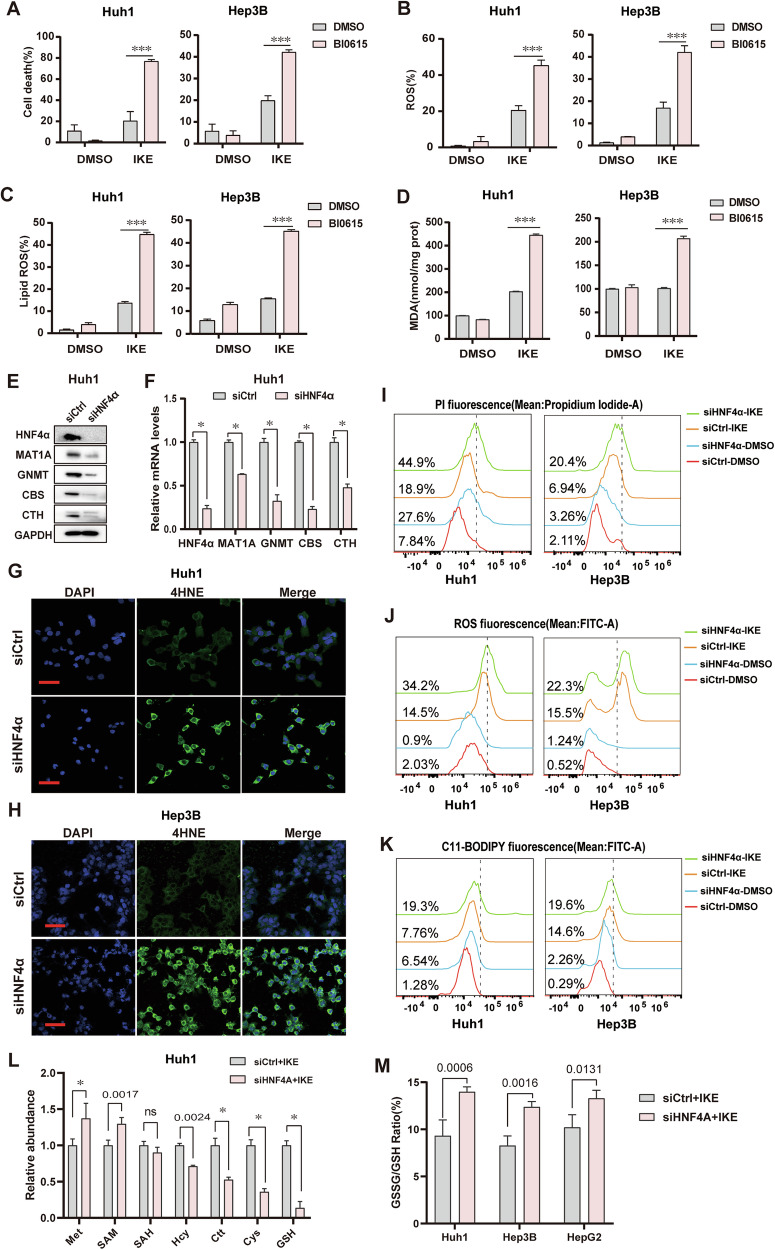
Targeting HNF4α sensitizes EC to IKE-induced ferroptosis. **A**–**D** Huh1 and Hep3B cells were incubated with DMSO or IKE (10 µM) in combination with the HNF4α inhibitor BI6015 (5 µM) for 24 h. Cell death was assessed by PI (2 µM) staining (**A**), total ROS by H2DCFDA (1 µM) (**B**), lipid ROS by C11-BODIPY (581/591, 2 µM) (**C**), and MDA levels (**D**) (n = 3), ***p < 0.0001. **E**, **F** After 48 h of siHNF4α transfection, HNF4α, MAT1A, GNMT, CBS, and CTH expression in Huh1 cells was measured by Western blot and RT-qPCR (n = 3), *p < 0.0001. Cells were transfected with siRNA for 24 h, treated with IKE (10 µM) for 10 h, and intracellular 4HNE levels were detected in Huh1 (**G**) and Hep3B (**H**) cells using immunofluorescence (green); nuclei were stained with DAPI (blue), scale bar = 50 µm (n = 3). Following 24 h of siRNA transfection, Huh1 and Hep3B cells were incubated with DMSO or IKE (10 µM) for 24 h, and cell death (**I**), total ROS (**J**), and lipid ROS (**K**) were measured. Fluorescence intensities were quantified using Cytek NL-CLC full-spectrum flow cytometry (n = 3). **L** LC-MS/MS quantification of methionine metabolism-related metabolites in Huh1 cells after siRNA transfection and IKE treatment (n = 3). ns indicates no significant difference, *p < 0.001. **M** GSSG/GSH ratio was measured in EC following siRNA transfection and 24 h IKE treatment (n = 3).

We treated three types of EC with BI6015, a specific inhibitor of HNF4α, and demonstrated that inhibition of HNF4α activity increased the sensitivity of EC to ferroptosis. This was evidenced by increased cell death induced by IKE (Fig. 5A, Fig. S6A), as well as elevated levels of intracellular total ROS (Fig. 5B, Fig. S6B), lipid ROS (Fig. 5C, Fig. S6C), and MDA levels (Fig. 5D, Fig. S6D). Moreover, knockdown of HNF4α by RNA interference directly resulted in downregulation of the major methionine metabolic enzymes at both the protein expression level and the relative mRNA level (Fig. 5E, F, Fig. S6E–H). Meanwhile, downregulation of HNF4α led to a significant increase in intracellular 4HNE levels (Fig. 5G, H, Fig. S6I). In addition, co-treatment with HNF4α knockdown and IKE enhanced the cytotoxic effects of IKE (Fig. 5I, Fig. S6J), as well as intracellular total ROS (Fig. 5J, Fig. S6K) and lipid peroxidation levels (Fig. 5K, Fig. S6L). Following co-treatment with HNF4α knockdown and IKE, the three types of EC showed increased levels of Met but reduced levels of Ctt, Cys, and GSH (Fig. 5L, Fig. S6M-N). Furthermore, the GSSG/GSH ratio also increased significantly (Fig. 5M). These observations indicate that HNF4α plays a significant role in conferring resistance to ferroptosis in EC, and that methionine metabolic enzymes are critical downstream effectors of HNF4α-mediated ferroptosis resistance.

### Targeting HNF4α mediates ferroptosis in vivo

**Fig. 6:**
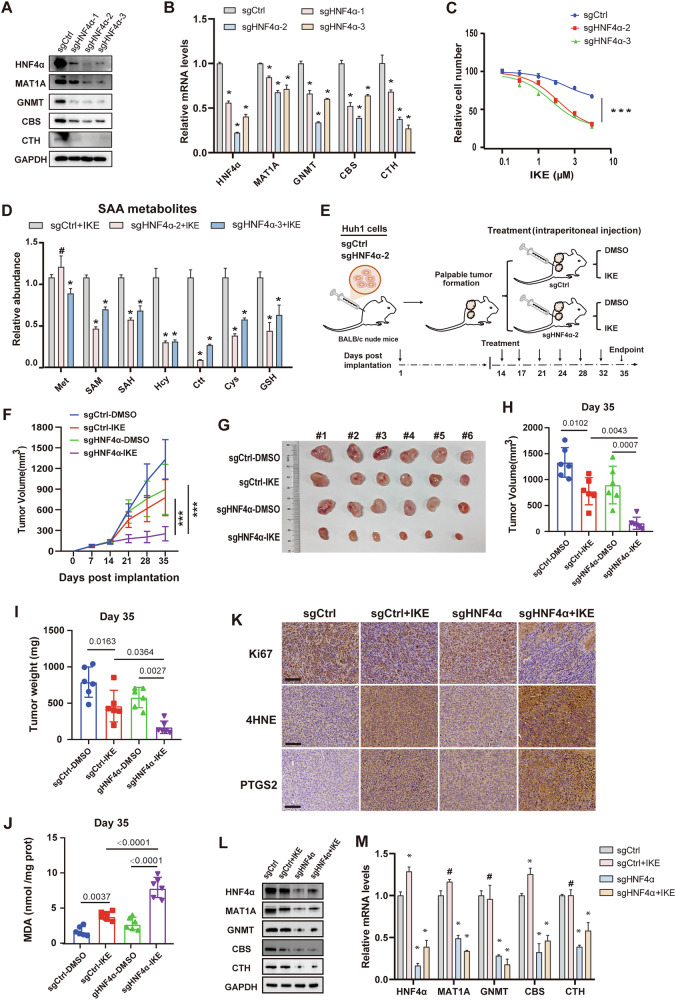
Targeting HNF4α suppresses tumor growth by enhancing ferroptosis in vivo. **A**, **B** Western blot and RT-qPCR analyses of HNF4α and major methionine metabolic enzymes expression levels in Huh1 cells transfected with control sgRNA (sgCtrl) or three sgRNAs targeting HNF4α (sgHNF4α-1, sgHNF4α-2, sgHNF4α-3). **C** Cell viability of Huh1-sgCtrl and Huh1-sgHNF4α-2/3 treated with IKE (10 µM) or DMSO, measured by MTS assay. **D** LC-MS/MS analysis of methionine metabolism-related metabolites in Huh1-sgCtrl and Huh1-sgHNF4α-2/3 cells (n = 3); # indicates no significant difference, *p < 0.005. **E** Schematic of in vivo experimental design. **F** Tumor volumes measured weekly (n = 6), ***p < 0.0001. **G** Tumor sizes on day 35 (n = 6). On day 35, tumor volumes (**H**), tumor weight (**I**) and MDA levels (**J**) were measured; expression of Ki67, 4HNE, and PTGS2 was assessed by immunohistochemistry (**K**), HNF4α and major methionine metabolic enzymes expression levels was assessed by Western blot (**L**), and RT-qPCR (**M**). Scale bar = 100 µm; # indicates no significant difference, *p < 0.005.

To further validate the above findings, we used CRISPR/Cas9 technology to generate HNF4α-knockdown Huh1 cells (Huh1-sgHNF4α) for xenograft experiments in vivo, with Huh1-sgCtrl serving as a negative control. Three independent sgRNA sequences targeting HNF4α were designed, and three stable Huh1-sgHNF4α cell lines (sgHNF4α-1, sgHNF4α-2, and sgHNF4α-3) were successfully established. As shown in Fig. 6A, B, compared with sgHNF4α-1, sgHNF4α-2 and sgHNF4α-3 demonstrated more pronounced HNF4α knockdown effects and induced a more significant downregulation of major methionine metabolic enzymes. Therefore, these two cell lines were selected for further investigation.

Decreased HNF4α expression in Huh1 cells increased sensitivity to IKE, consistent with the in vitro results presented in Fig. 5 (Fig. 6C). Similarly, upon treatment of Huh1-sgCtrl and Huh1-sgHNF4α cells with IKE, HNF4α depletion increased cell death (Fig. S7A), total ROS (Fig. S7B), and lipid ROS levels (Fig, S7C). Metabolite profiling of Huh1-sgCtrl and Huh1-sgHNF4α-2 cells revealed significant differences, with KEGG enrichment analysis indicating that metabolites downregulated in sgHNF4α-2 were enriched in cysteine and methionine metabolism (Fig. S7D-E). As shown in Fig. 6D, intracellular levels of SAM, SAH, Hcy, Ctt, Cys, and GSH were significantly reduced in HNF4α-depleted Huh1 cells, confirming that the two HNF4α-stably deleted cell lines exhibited markedly increased sensitivity to IKE.

For in vivo studies, sgHNF4α-2 Huh1 cells were used to establish a xenograft model in immunodeficient BALB/c nude mice. A total of 1 × 10^7^ Huh1-sgCtrl or Huh1-sgHNF4α-2 cells were injected subcutaneously, and IKE (30 mg/kg) was administered twice weekly for three weeks, starting on day 14 when tumors reached an average volume of 80–100 mm^3^ (Fig. 6E). Tumor volumes (Fig. 6F) and body weights (Fig. S7F) were monitored weekly. No significant body weight loss was observed, indicating the treatment was well tolerated. On day 35, HNF4α deletion significantly enhanced IKE-induced tumor suppression compared with controls (Fig. 6G), as evidenced by reduced tumor volume and weight (Fig. 6H, I), increased MDA levels (Fig. 6J), and elevated intratumoral 4HNE and prostaglandin-endoperoxide synthase 2 (PTGS2) levels (Fig. 6K), all established biomarkers of ferroptosis. Furthermore, the protein (Fig. 6L) and mRNA levels (Fig. 6M) of HNF4α and major methionine metabolic enzymes in tumor tissues were consistent with those observed in cells prior to xenotransplantation, indicating that HNF4α continues to regulate methionine metabolic enzyme expression in vivo. Collectively, these results provide robust evidence that targeting HNF4α enhances ferroptosis-mediated tumor suppression in vivo.

## Discussion

HCC, accounting for approximately 91.5% of primary liver cancers, remains one of the most aggressive malignancies with a poor prognosis, representing a major public health challenge worldwide [1, 2]. For many years, induction of tumor cell apoptosis has been considered the primary therapeutic approach for various cancers. However, the clinical efficacy of apoptosis-based therapies has been limited [32–34]. Thus, there is an urgent need to identify novel cancer treatments that act through mechanisms beyond apoptosis. Ferroptosis, a regulated form of cell death distinct from apoptosis, has gained increasing attention in tumor biology and cancer therapy research [5, 6]. Consequently, targeting ferroptosis has emerged as a promising anticancer strategy [35, 36]. Nonetheless, tumor heterogeneity—manifested by the coexistence of diverse cell subtypes within the same HCC tissue—introduces variability in drug sensitivity and partially limits the broad application of ferroptosis-based therapies across different HCC subtypes [37–39].

EMT is widely recognized as a key driver of tumor metastasis [24]. Numerous studies have demonstrated that cancer cells exhibiting a mesenchymal phenotype are particularly sensitive to ferroptosis inducers, whereas epithelial cancer cells display relative resistance [23, 40]. In this study, we classified 25 HCC cell lines from the CCLE database into epithelial and mesenchymal subtypes based on established EMT markers (Fig. 1A). Functional assays confirmed that EC are less sensitive to the ferroptosis inducer IKE than MC, consistent with previous reports (Fig. 1B, C). Investigating the mechanisms underlying EC resistance to ferroptosis may provide novel insights to advance ferroptosis-based therapies for liver cancer. Transcriptomic and metabolomic analyses revealed that the differentially expressed genes and metabolites in EC were significantly enriched in the cysteine and methionine metabolism pathways (Fig. 1D, H). Previous studies have established that cysteine and methionine metabolism is closely linked to tumorigenesis, and that methionine deprivation can enhance tumor ferroptosis [41–43].

Notably, when cystine availability is limited, methionine metabolism can be activated to generate cysteine de novo from methionine [16]. Our findings extend this concept by identifying a subtype-specific metabolic adaptation in HCC: EC maintain intracellular cysteine pools via dual mechanisms—cystine import through the xCT transporter and de novo synthesis via the methionine metabolism pathway. This metabolic flexibility sustains glutathione biosynthesis, thereby protecting EC from ferroptosis under cystine-limiting conditions (Fig. 2). Importantly, disrupting this compensatory pathway—either through RNA interference targeting MAT1A, GNMT, CBS, or CTH, or by pharmacological inhibition—abolished ferroptosis resistance in EC. These results highlight the therapeutic potential of targeting methionine metabolism to overcome ferroptosis resistance in EC.

We next sought to identify the transcriptional regulator governing methionine metabolism in EC. Through correlation analysis and transcription factor enrichment screening, HNF4α emerged as a candidate upstream regulator [43]. HNF4α, a liver-enriched nuclear receptor, orchestrates the expression of genes involved in hepatocyte differentiation, drug metabolism, and liver-specific functions. Dysregulation of HNF4α has been implicated in various liver pathologies, and accumulating evidence indicates that its downregulation is associated with EMT progression in HCC. Consistently, we observed that HNF4α expression was significantly higher in EC and primary tumors compared with mesenchymal cells and metastatic lesions. Furthermore, HNF4α levels inversely correlated with mesenchymal markers (Vimentin, CD44) (Fig. S4E) and EMT-associated transcription factors (Twist1, SNAI1), supporting its role in maintaining the epithelial phenotype (Fig. S4D). Mechanistically, bioinformatic analysis predicted HNF4α-binding motifs in the promoter regions of MAT1A, GNMT, CBS, and CTH. ChIP-qPCR and luciferase reporter assays confirmed that HNF4α directly binds to and transactivates these promoters. Functionally, knockdown or pharmacological inhibition of HNF4α downregulated the expression of methionine metabolism enzymes, depleted intracellular glutathione, and sensitized EC to ferroptosis both in vitro and in vivo. Collectively, these results establish a previously unrecognized HNF4α-methionine metabolism-ferroptosis resistance axis in EC.

**Fig. 7:**
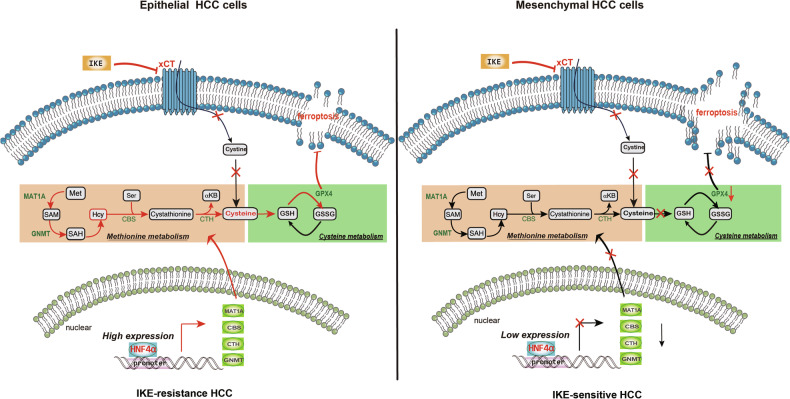
Schematic representation of ferroptosis mediated by targeting HNF4α. Left：In epithelial HCC cells, high expression of HNF4α transcriptionally upregulates key enzymes involved in methionine metabolism, thereby activating the methionine metabolic pathway. This activation compensates for the cysteine deficiency caused by IKE-mediated inhibition of cystine uptake, and the resulting cysteine metabolic pathway subsequently suppresses the accumulation of intracellular lipid peroxides. Right：In contrast, mesenchymal HCC cells exhibit low HNF4α expression, leading to suppression of the methionine metabolic pathway. Concomitantly, IKE inhibits cystine uptake, resulting in decreased cysteine levels, accumulation of intracellular lipid peroxides, and ultimately the induction of ferroptosis.

In summary, this study elucidates a novel mechanism by which EC evade ferroptosis via HNF4α-driven methionine metabolism (Fig. 7). By transcriptionally upregulating MAT1A, GNMT, CBS, and CTH, HNF4α enhances the conversion of methionine to cysteine, thereby sustaining glutathione synthesis and maintaining redox homeostasis under cystine-limited conditions. These findings provide a mechanistic explanation for the differential ferroptosis sensitivity observed between HCC subtypes and identify HNF4α and its downstream metabolic enzymes as potential therapeutic targets.

Nevertheless, several limitations should be acknowledged. First, the regulation of gene promoters rarely relies on a single transcription factor; rather, it is mediated through a complex network comprising activators, repressors, co-regulators, and epigenetic modifications. Future studies are required to systematically define the hierarchical and synergistic relationships among these transcriptional regulators in this context. This study provides substantial evidence for the central role of HNF4α and establishes the groundwork for further mapping of the complete regulatory network. Second, the metabolomics data presented represent steady-state measurements; while they strongly suggest that methionine fuels glutathione synthesis, the precise metabolic flux through the methionine pathway under cystine-limited conditions remains to be quantified using isotope tracing. Third, our in vivo validation employed cell line-derived xenografts, which may not fully recapitulate the metabolic heterogeneity of human HCC. Taken together, these limitations indicate that our findings should be regarded as indicative of a novel regulatory mechanism and provide a foundation for future studies aimed at deeper mechanistic dissection and clinical translation.

## Conclusions

In conclusion, our study systematically demonstrates that overexpression of HNF4α activates the methionine metabolic pathway, thereby promoting intracellular cysteine replenishment and glutathione synthesis under cystine-limited conditions, ultimately enhancing the resistance of epithelial HCC cells to ferroptosis. This mechanism highlights a central role for HNF4α in modulating ferroptosis sensitivity across HCC subtypes through the transcriptional regulation of key methionine metabolic enzymes, including MAT1A, GNMT, CBS, and CTH. Our findings not only reveal a novel molecular pathway underlying ferroptosis resistance in epithelial HCC subpopulations but also identify this regulatory axis as a potential therapeutic target. Moreover, these results provide a conceptual framework for the development of ferroptosis-based combination therapies, particularly for HCCs exhibiting an epithelial phenotype.

## Methods

### Cluster analysis of hepatocellular carcinoma cells

To distinguish epithelial and mesenchymal hepatocellular carcinoma (HCC) cells, cluster analysis was performed on the expression data of 25 HCC cell lines obtained from the Broad Institute Cancer Cell Line Encyclopedia (CCLE) using the R function hclust. Cell lines were clustered based on the expression of mesenchymal markers (CD44 and vimentin), epithelial markers (CDH1, KRT8, and KRT18), and transcription factors regulating epithelial-mesenchymal transition (EMT) (ZEB1 and TWIST1) as reported in previous studies. Z-scores were calculated for log_2_(RPKM + 1) values to standardize the data for cluster analysis. Pairwise relationships between samples were quantified using Euclidean distance as a similarity metric. Hierarchical clustering was conducted using the hclust function with the Ward.D2 method to generate a cluster dendrogram. A binary hierarchical cluster tree was then constructed using the color_branches function, which relies on the cutree function from the R package dendextend. Finally, clusters were visualized with the Heatmap function in the R package ComplexHeatmap.

### Cell culture

Six HCC cell lines—Hep3B, HepG2, Huh1, SNU387, SNU182, and SK-HEP-1—were obtained from Tianjin Institute of Pharmaceutical Research Co., Ltd. All cell lines were originally sourced from the American Type Culture Collection (ATCC).

Huh1 and SK-HEP-1 cells were maintained in DMEM (Gibco) supplemented with 10% fetal bovine serum (FBS) (Gibco). Hep3B and HepG2 cells were maintained in MEM (Gibco) supplemented with 1× Non-Essential Amino Acids (NEAA) (Gibco) and 10% FBS. SNU182 and SNU387 cells were maintained in RPMI-1640 (Gibco) with 10% FBS. All cells were cultured under standard conditions at 37 °C in a humidified atmosphere containing 5% CO_2_.

### Cystine and methionine limitation experiments

Four types of medium were prepared as follows: DMEM (Gibco, 21013-024) supplemented with 10% FBS, 130 μM methionine, 160 μM cystine, 1× Glutamine, and sodium pyruvate was used as complete medium (CM). CM devoid of cystine was used as cystine-restricted medium (CRM), CM devoid of methionine as methionine-restricted medium (MRM), and CM devoid of both methionine and cystine as double-restricted medium (DRM). A total of 2000–4000 HCC cells per well were seeded into E-Plate View 96-well plates (Agilent). After 24 h culture in complete medium (DMEM or RPMI-1640 with 10% FBS), the medium was replaced with the four experimental media (n = 3 replicates per group). Cell proliferation was continuously monitored using the xCELLigence Real-Time Cell Analysis (RTCA) eSight system (Agilent). The RTCA eSight system is a microelectronic biosensor platform that measures electrical impedance via a gold-coated, crossed digital microelectrode array on a dedicated 96-well E-Plate. Impedance measurements were expressed as the Cell Index (CI), providing quantitative information on cell morphology, number, and viability. Images were captured every 30 min, and CI values were recorded every 15 min. The growth of cells under each medium condition was observed in real time for up to one week.

### RNAi and CRISPR/Cas9 mediated gene knockdown

siRNA transfection: Approximately 10 nM of siRNA was incubated in 200 µL Opti-MEM (Gibco) mixed with 2.5 µL Lipofectamine RNAiMAX (ThermoFisher) for 15 min. The mixture was then added to 1 mL of cell suspension, with 150,000 HCC cells seeded per well in 12-well plates. Cells were analysed 24 h post-transfection. siRNAs targeting individual methionine metabolism genes were purchased from MCE (CTH, HY-RS03296; CBS, HY-RS02007; GNMT, HY-RS05569; MAT1A, HY-RS08166). siRNA targeting HNF4α (SC-35573) was obtained from Santa Cruz Biotechnology.

CRISPR/Cas9 knockdown: To knock down HNF4α, three sgRNAs were designed (sgRNA-1: TACGGTGCCTCGAGCTGTGA; sgRNA-2: GCAATGACTACATTGTCCCT; sgRNA-3: CCAAGGGGCTGAGCGATCCA) and synthesised by Sangon Biotech. Each sgRNA was cloned into the PX459 vector and transfected separately into HCC cells using Lipofectamine 3000 (ThermoFisher). Transfected cells were selected with puromycin (Solarbio, P8230) to establish stable knockdown lines.

### LC-MS/MS

Culture medium was aspirated from approximately 10^7^ cells per sample. Proteins were precipitated with 800 µL cold methanol, and metabolites were extracted by high-speed centrifugation for subsequent analysis. Metabolite separation and detection were performed using high-performance liquid chromatography (HPLC) (Thermo Scientific^TM^ Vanquish^TM^) coupled with a triple quadrupole mass spectrometer (Thermo Scientific^TM^ TSQ Altis^TM^ Plus). Separations were achieved using hydrophilic interaction liquid chromatography (HILIC) with an ACQUIY UPLC BEH Amide column (2.1 mm × 100 mm, 1.7 µm, Waters). Mobile phase A consisted of 25 mM ammonium hydroxide and 25 mM ammonium acetate in water, while acetonitrile was used as mobile phase B.

### Transcriptome analysis

Transcriptome sequencing (n = 3) of Huh1 and SNU182 cell lines was performed by Shanghai Applied Protein Technology Co., Ltd. Raw sequencing reads were trimmed using fastp with parameters --n_base_limit 5 --average_qual 20 -l 50, followed by alignment to the GRCh38.p13 reference genome using STAR in GeneCounts mode. Differential expression analysis was performed using the limma-trend method, and genes with |log_2_ FC | > 2 and FDR < 0.05 were defined as differentially expressed. Expression levels of mesenchymal and epithelial markers, as well as EMT-regulating transcription factors, were visualised using the R package pheatmap. Functional enrichment analysis was conducted using the R package clusterProfiler and visualized with ggplot2 and enrichplot.

### Metabolomic analysis

Metabolomic profiling (n = 5) of Huh1 and SNU182 cells was performed by Shanghai Applied Protein Technology Co., Ltd. Data analysis was conducted using the R package MetaboAnalystR (version 4.0.0) in the Pathway Analysis module. Metabolite peak tables were filtered based on QC samples with a relative standard deviation (RSD) cutoff of 80 and an interquartile range (IQR) cutoff of 10. Data were normalised row-wise to the reference QC group, log-transformed (LogNorm), and scaled using AutoNorm. Principal component analysis (PCA) was performed for quality control using the PCA.Anal function. QC samples and outliers were subsequently removed. KEGG functional enrichment analysis was conducted using the PerformPSEA function, which employs the mummichog algorithm.

### Immunoblotting

Protein samples were analysed using the following antibodies: GNMT (18790-1-AP, 1:1000, Proteintech), MAT1A (67408-1-Ig, 1:1000, Proteintech), CBS (ab140600, 1:1000, Abcam), CTH (12217-1-AP, 1:1000, Proteintech), HNF4α (MA5-14891, 1:1000, ThermoFisher Scientific), GAPDH (60004-1-Ig, 1:1000, Proteintech), HRP-conjugated Goat Anti-Mouse IgG (SA00001-1, 1:2000, Proteintech), and HRP-conjugated Goat Anti-Rabbit IgG (SA00001-2, 1:2000, Proteintech).

### RT-qPCR

Total RNA was extracted using the TransZol Up Plus RNA Kit (ER501-01-V2, Transgen). cDNA was synthesised with the Transcriptor First Strand cDNA Synthesis Kit (Roche) on a T100 Thermal Cycler (BIO-RAD). Quantitative PCR (qPCR) was performed using FastStart Universal SYBR Green Master (ROX) (Roche) on the Roche LightCycler 480 II Real-Time System. Data were normalised to GAPDH, and fold changes relative to the control group were calculated using the 2^–ΔΔCT^ method. Primer sequences are listed in Supplementary Table 1.

### Luciferase assay

Publicly available Chromatin Immunoprecipitation (ChIP)-seq data from the ENCODE project for HNF4α in human liver or HepG2 cells indicated the promoter regions of MAT1A, GNMT, CTH, and CBS. The transcription start site was defined as +1 bp. The promoter regions were defined as follows: MAT1A, −2000 to −1 bp; GNMT, −2000 to −1 bp; CTH, −1900 to +100 bp; CBS, +8620 to +10,620 bp (UCSC Genome Browser). These sequences were cloned into the pGL4.20 [luc2 Puro] vector (Promega). Deletion mutants, defined as removal of a 200-bp fragment containing the HNF4α peak, were generated by Fenghui Shengwu Co., Ltd. HNF4α cDNA was cloned into the pcDNA3.1 vector. SNU182 cells were co-transfected with pcDNA3.1-HNF4α or empty vector, the Renilla luciferase plasmid (pRL-SV40-N, Promega), and either the wild-type (WT) or deletion mutant (Mut) promoter constructs using Lipofectamine 3000 (ThermoFisher Scientific). Luciferase activity was measured 24 h post-transfection using the Dual-Luciferase Reporter Assay System (Promega) and normalised to Renilla luciferase activity.

### ROS analysis

Intracellular reactive oxygen species (ROS) were detected using 1 µM H2DCFDA (ThermoFisher Scientific, D399), and lipid ROS were detected using 2 µM C11-BODIPY (581/591) (ThermoFisher Scientific, D3861). Cells were incubated in the dark at 37 °C for 30 min with the respective probes. Fluorescence was analysed using a Cytek NL-CLC full-spectrum flow cytometer.

### Lipid peroxidation assay

Intracellular malondialdehyde (MDA) levels were quantified using a lipid peroxidation assay kit (Solarbio, BC0025). Cells were homogenised in MDA extraction buffer on ice and lysed by ultrasound (200 W power, 3 s pulses, 10 s intervals, repeated three times). The lysates were centrifuged at 10,000 × g for 15 min at 4 °C. MDA was reacted with thiobarbituric acid (TBA) to form MDA–TBA adducts, and the intensity of the reaction, measured spectrophotometrically, is directly proportional to the MDA concentration.

### Cell viability and death assay

Cell viability was quantified using the CellTiter 96^®^ AQueous One Solution Cell Proliferation Assay (MTS) (G3582, Promega). Cells were seeded in 96-well plates and treated with the specified compounds for predetermined durations. Following treatment, MTS working solution was added, and plates were incubated for 1–4 h at 37 °C with 5% CO₂. Absorbance at 490 nm was measured, with viable cell number positively correlating with absorbance.

To assess cell death, cells were incubated with 2 µM Propidium Iodide (PI) (Beyotime) at 37 °C for 10 min in the dark. Cells were washed three times with PBS, harvested, and resuspended in PBS. Fluorescence was analysed using a Cytek NL-CLC full-spectrum flow cytometer.

### Immunocytofluorescence

Cells were fixed on slides with 4% paraformaldehyde. Oxidative stress was assessed by immunofluorescent staining of 4-hydroxynonenal (4HNE). Slides were incubated overnight at 4 °C with 4HNE Monoclonal Antibody (12F7) (Invitrogen, MA5-27570, 1:100), followed by incubation with FITC-conjugated Goat anti-Mouse IgG (H + L) (ABclonal, AS001-100UL, 1:200) for 1 h. Slides were counterstained with DAPI antifade solution (Solarbio, S2110) and examined using fluorescence microscopy.

### Chromatin immunoprecipitation (ChIP)

ChIP was performed using the Pierce Magnetic ChIP Kit (Thermo Scientific). Briefly, 4 × 10^6^ cells were treated with 1% fresh formaldehyde. Cells were lysed, enzymatically digested, and sonicated to produce chromatin fragments of 150–1000 bp. Ten percent of the chromatin was reserved as input control. The remaining chromatin was immunoprecipitated with anti-HNF4α antibodies (ThermoFisher Scientific, MA1-199, 1:100). Protein A/G magnetic beads were added and incubated with the chromatin-antibody complex for 2 h at 4 °C. Chromatin was eluted, crosslinks reversed, and DNA purified using a DNA cleanup column. Immunoprecipitated and input DNA samples were analyzed by RT-qPCR on the Roche LightCycler 480 II Real-Time System. Primer sequences are listed in Supplementary Table 1.

### Xenograft experiment

Experimental mice were housed in a controlled environment at 19–23 °C with ad libitum access to food and water and a 12 h light/dark cycle.

1 × 10⁷ sgCtrl-Huh1 or sgHNF4α-Huh1 cells were subcutaneously xenografted into both flanks of 5-week-old male BALB/c nude mice (Charles River Laboratories). Tumor growth and overall health were monitored twice weekly. Mice were weighed, and tumor volumes were measured weekly. When mean tumor volumes reached 80–100 mm³ (~2 weeks post-implantation), mice were randomly assigned to two treatment groups (n = 8 per group), ensuring that the average weight was comparable between groups to minimize selection bias. One group received intraperitoneal control solvent and the other received intraperitoneal IKE (30 mg/kg). Treatments were administered twice weekly for three weeks. Any mice that lost more than 20% of the initial weight during treatment, or tumor diameter exceeded 15 mm, were immediately eliminated from this sample and were not counted in the statistical analysis. Tumor length and width were measured using calipers, and volume was calculated using the formula V = length × width^2^ / 2.

Experiments were performed in accordance with the guidelines of the Animal Ethics Committee of Tianjin Tiancheng New Drug Evaluation (Approval No. Co.2021-0008). Mice were anaesthetised with isoflurane delivered via an anesthetic ventilator with oxygen (induction at 2.5%, maintenance at 1.5%).

### Immunohistochemistry

Formalin-fixed xenograft tumor sections were prepared using a cryostat (Leica, CM1950). Sections were incubated overnight at 4 °C with 4HNE Monoclonal Antibody (12F7) (MA5-27570, 1:100, Invitrogen), Ki67 Polyclonal Antibody (27309-1-AP, 1:100, Proteintech), or PTGS2 Polyclonal Antibody (27308-1-AP, 1:100, Proteintech). HRP-conjugated Goat Anti-Mouse IgG (H + L) (SA00001-1, 1:200, Proteintech) or HRP-conjugated Goat Anti-Rabbit IgG (H + L) (SA00001-2, 1:200, Proteintech) was used as the secondary antibody for 1 h at room temperature. Sections were visualised with 3,3′-diaminobenzidine (DAB) Liquid Substrate System (D7304, Sigma) for 30 s at room temperature. After washing, sections were counterstained with hematoxylin, dehydrated through graded ethanol, cleared in xylene, mounted, and examined microscopically.

### Glutathione disulfide (GSSG)/glutathione (GSH) ratio

Following treatment, cells were digested with 0.25% trypsin, washed twice with PBS, and lysed with NP-40 to obtain cell homogenates. Lysates were centrifuged at 14,000 × *g* for 15 min at 4 °C, and the supernatant was used to determine the intracellular GSSG/GSH ratio using the GSH/GSSG Ratio Detection Assay Kit (Abcam, ab138881), according to the manufacturer’s instructions.

### Statistical analysis

Outcome assessment was performed by an independent investigator who coded and analyzed all data without knowledge of the group assignments. All data are presented as the mean ± SD of three independent replicates. Statistical analyses were performed using GraphPad Prism (version 8.0.0). Each group of experiments was performed in at least three replicates, and all data followed a normal distribution. Two-way analysis of variance (ANOVA) followed by Dunnett’s multiple comparison test was used to evaluate statistical significance, and significant differences were defined with *p* values less than 0.05.

## Supplementary information

Uncropped western blots

Supplementary

## Data Availability

The list of transcription factors was obtained from the website (https://guolab.wchscu.cn/AnimalTFDB4//?#/Download). The differential expression of tumor and metastatic tissues in the liver cancer of HNF4α and key genes which participated in methionine metabolism were performed on the website TNMplot (https://tnmplot.com/analysis/). TCGA RNA-seq data were obtained from website (http://gdac.broadinstitute.org/runs/stddata__2016_01_28/data/LIHC/20160128/). CCLE RNA-seq data of human liver cancer cell lines were obtained from website (https://portals.broadinstitute.org/ccle/data). Noncommercial materials will be provided upon reasonable request.
