# Supplementary material for: Activation of methionine metabolism mediated by HNF4α confers ferroptosis resistance in hepatocellular carcinoma
Source: Cell Death Discov. 2026 May 26;12:316. doi: 10.1038/s41420-026-03165-0 (PMC13385955; doi:10.1038/s41420-026-03165-0)
Supplement: Supplementary file 1 — Uncropped western blots [file 41420_2026_3165_MOESM1_ESM.docx]

**Uncropped western blots**

**Fig 1F**

**Western blot was performed to detected the expression levels of** **MAT1A, GNMT, CBS and CTH in epithelial and mesenchymal hepatocellular carcinoma cells**

**
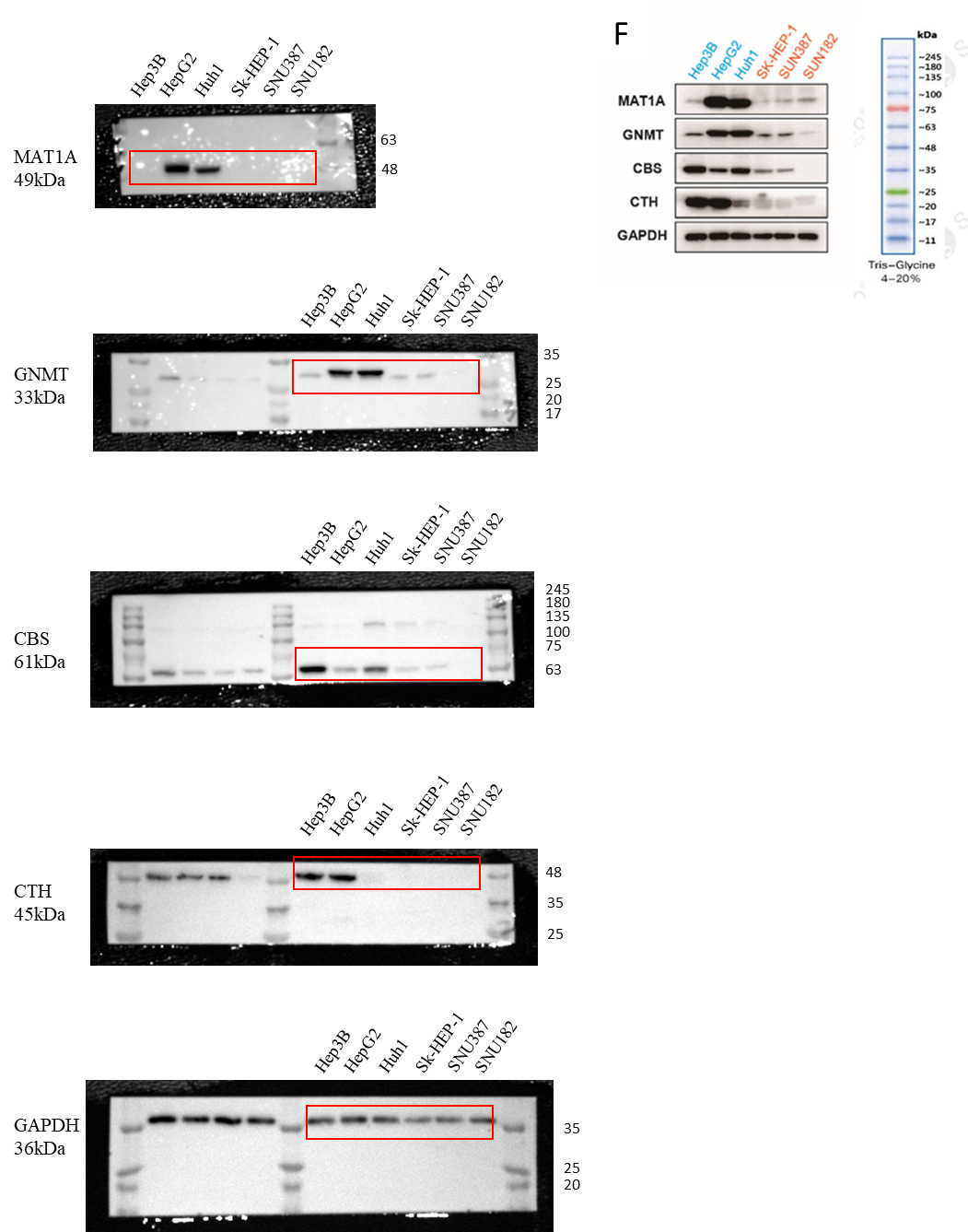
**

**Fig 4C**

**Western blot was performed to detected the expression levels of HNF4α in EC (HepG2, Huh1, Hep3B) and MC (SK-HEP-1, SNU182, SNU387).**

**
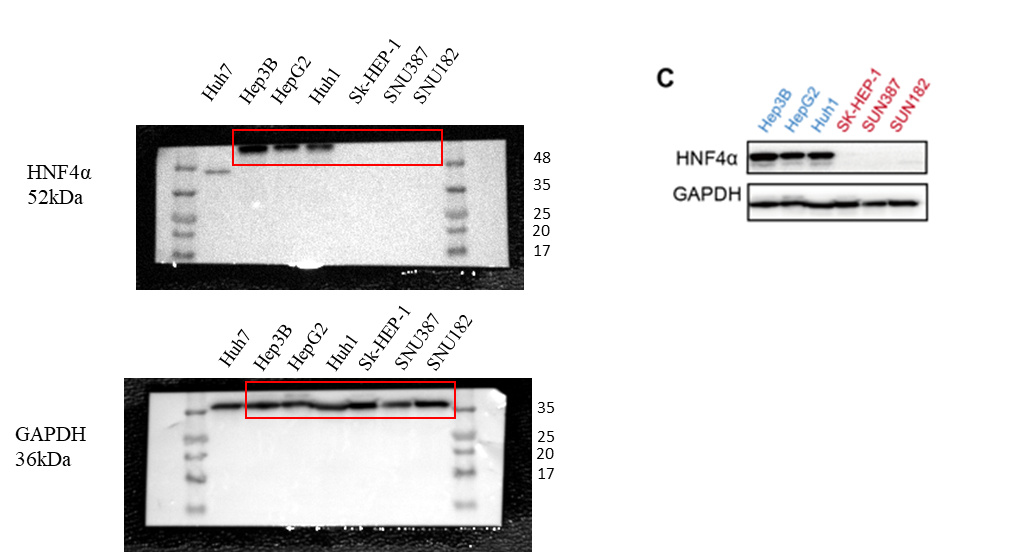
**

**Fig 5E**

**Western blot was performed to detect the expression levels of HNF4α, MAT1A, GNMT, CBS and CTH in Huh1 cells**

**
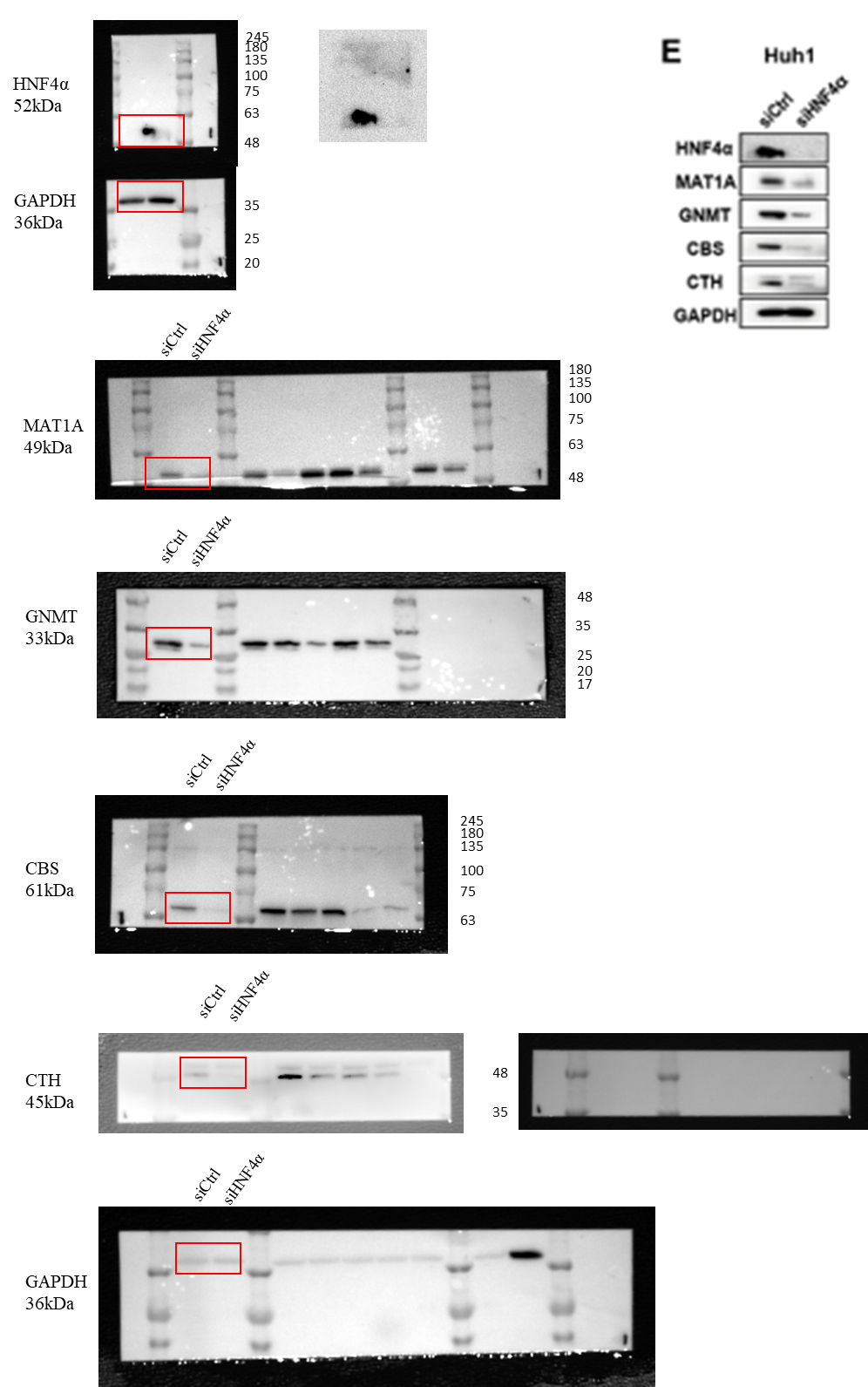
**

**Fig 6A**

**Western blot analysis in Huh1 cells expressing control small guide RNA (sgCtrl) or three independent sgRNA sequences targeting the HNF4α gene (sgHNF4α-1, sgHNF4α-2, and sgHNF4α-3).**

**
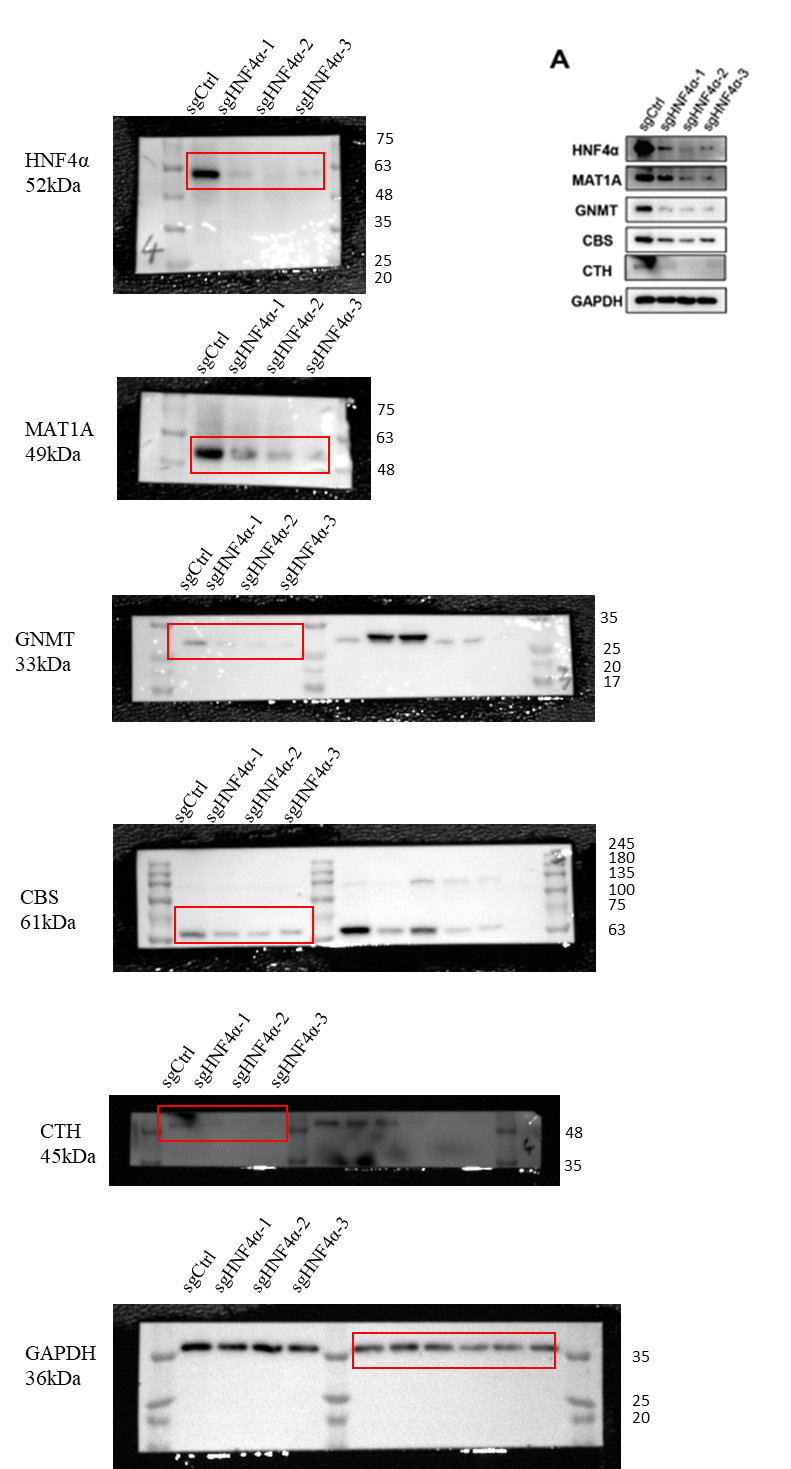
**

**Supplementary Fig. 6L**

**Western blot was performed to detect the expression of HNF4α, MAT1A, GNMT, CBS and CTH in tumor tissue.**

**
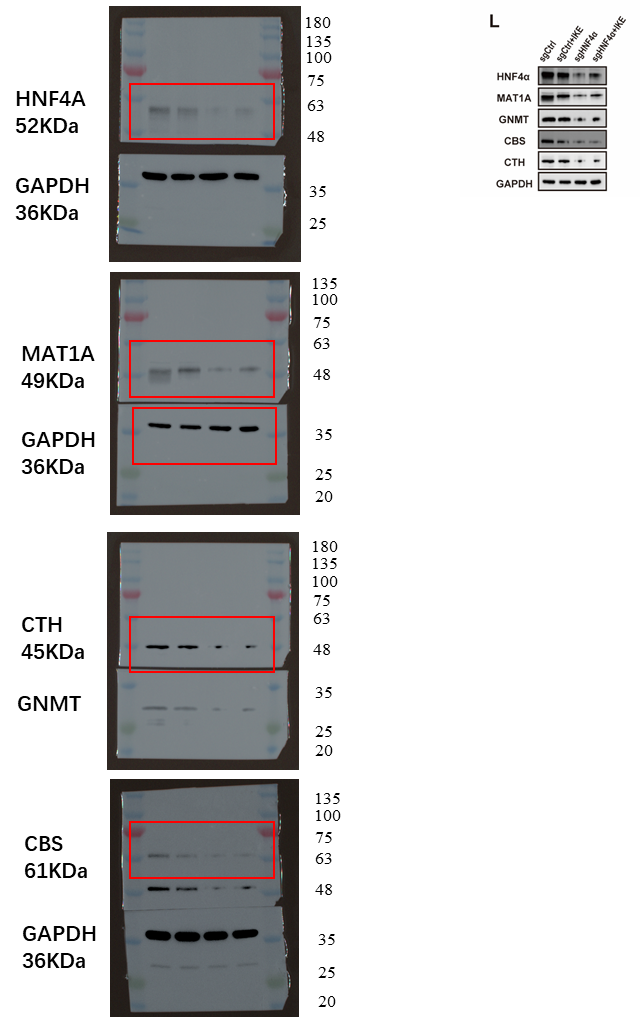
**

**Supplementary Fig. 6E**

**Western blot was performed to detect the expression of HNF4α, MAT1A, GNMT, CBS and CTH in Hep3B cells**

**
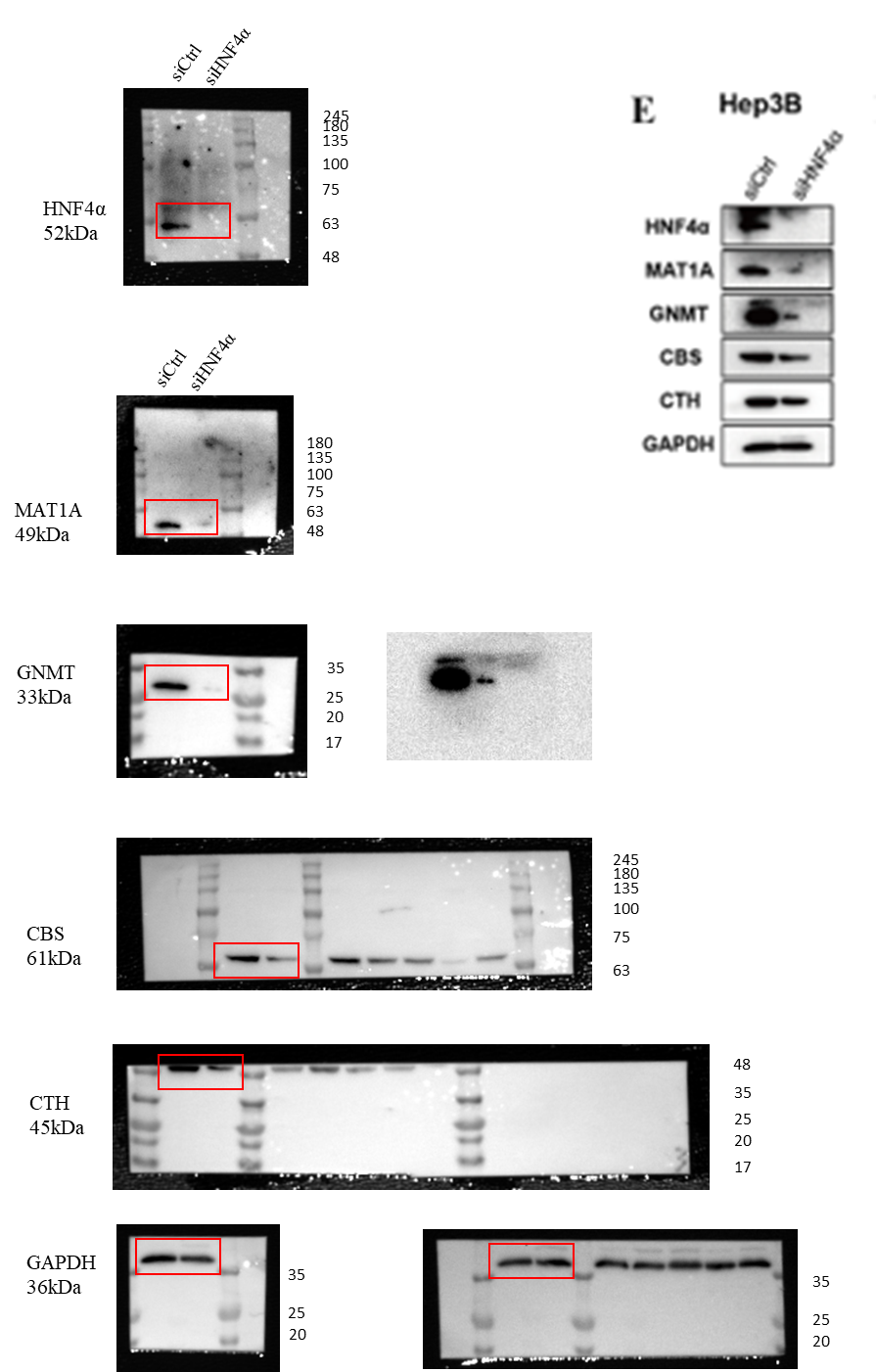
**

**Supplementary Fig. 6G**

**Western blot was performed to detect the expression of HNF4α, MAT1A, GNMT, CBS and CTH in HepG2 cells**

**
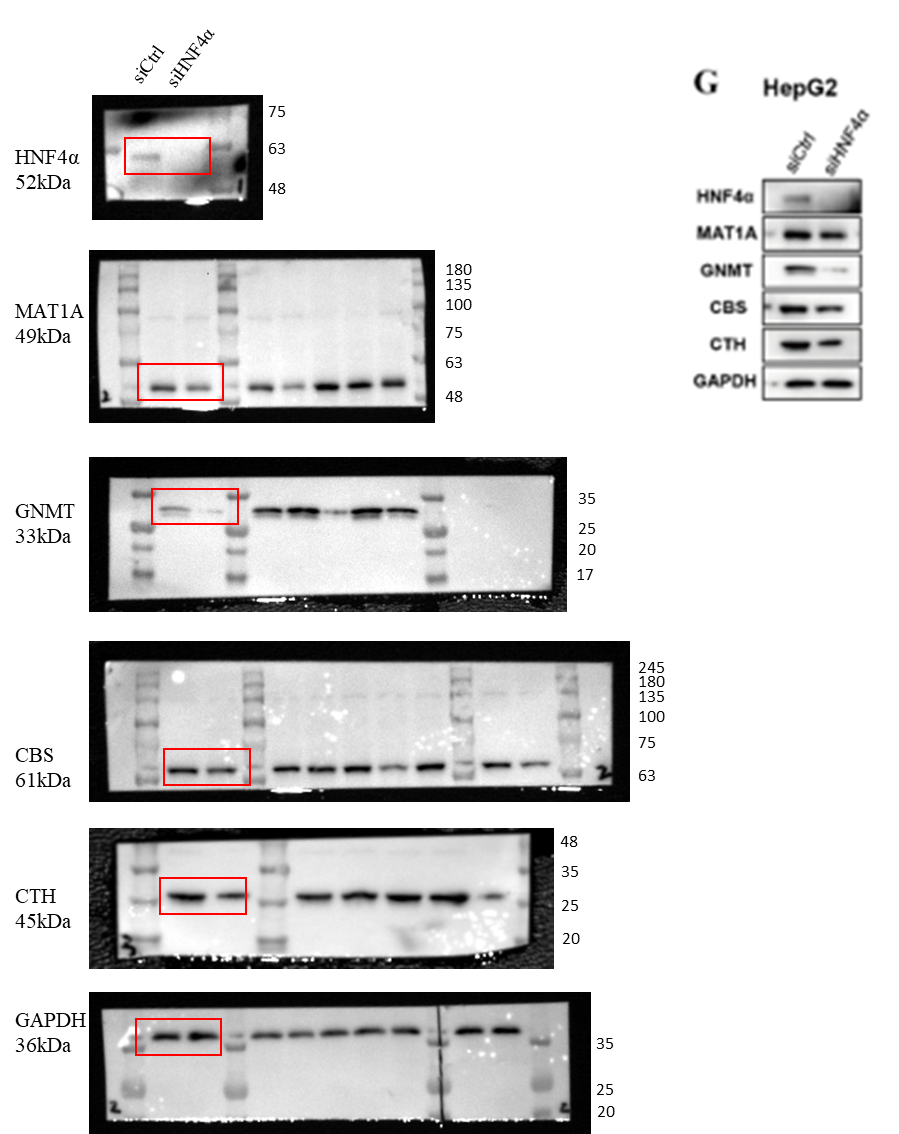
**
