# Supplementary material for: Activation of methionine metabolism mediated by HNF4α confers ferroptosis resistance in hepatocellular carcinoma
Source: Cell Death Discov. 2026 May 26;12:316. doi: 10.1038/s41420-026-03165-0 (PMC13385955; doi:10.1038/s41420-026-03165-0)
Supplement: Supplementary file 2 — Supplementary [file 41420_2026_3165_MOESM2_ESM.docx]

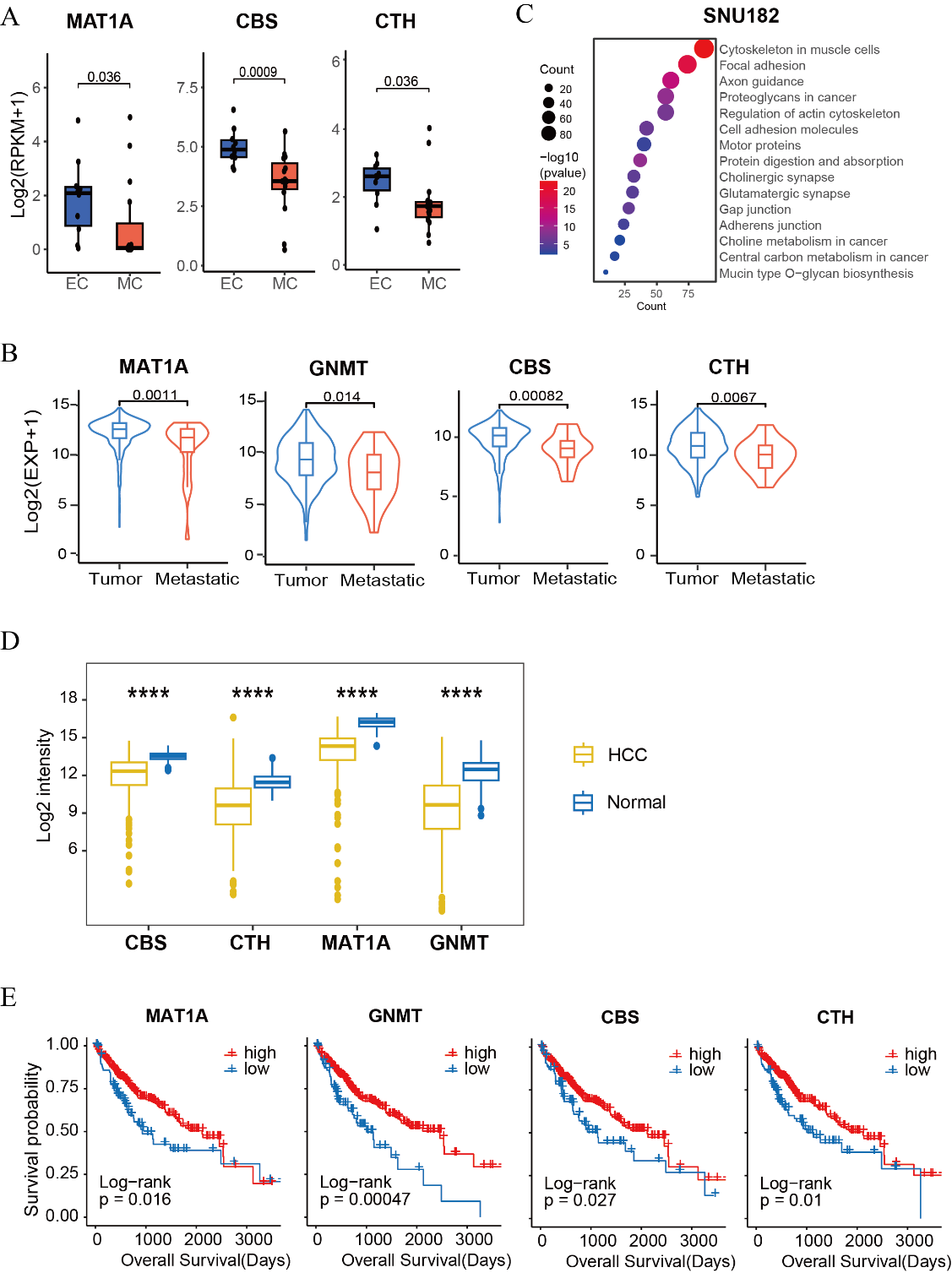


**Supplementary Fig. 1 Expression and functional analysis of key methionine metabolic enzymes from transcriptome data.**

(A) The expression levels of MAT1A, CBS and CTH were significantly higher in EC than MC. We analyzed the mRNA levels of these genes using 25 hepatocellular carcinoma cells from the CCLE database (n=10 epithelial, 15 mesenchymal). (B) The major methionine metabolic enzymes in tumor and metastatic tissues of liver cancer, the data were obtained from TNMplot website and plotted with R package ggplot2. (C) KEGG enrichment analysis of highly expressed genes in SNU182 cells. (D) The expression of major methionine metabolic enzymes is reduced in HCC tumors. The TCGA RNA-seq gene expression data, processed for n=50 normal and n=373 tumor LIHC samples, were downloaded from the TCGA data portal. (E) High expression levels of major methionine metabolic enzymes are associated with increased survival in HCC patients (n=373). Survival distributions were visualized by Kaplan-Meier survival curves, which were performed by classifying the expression of samples by lower quartile of the corresponding gene expression values.


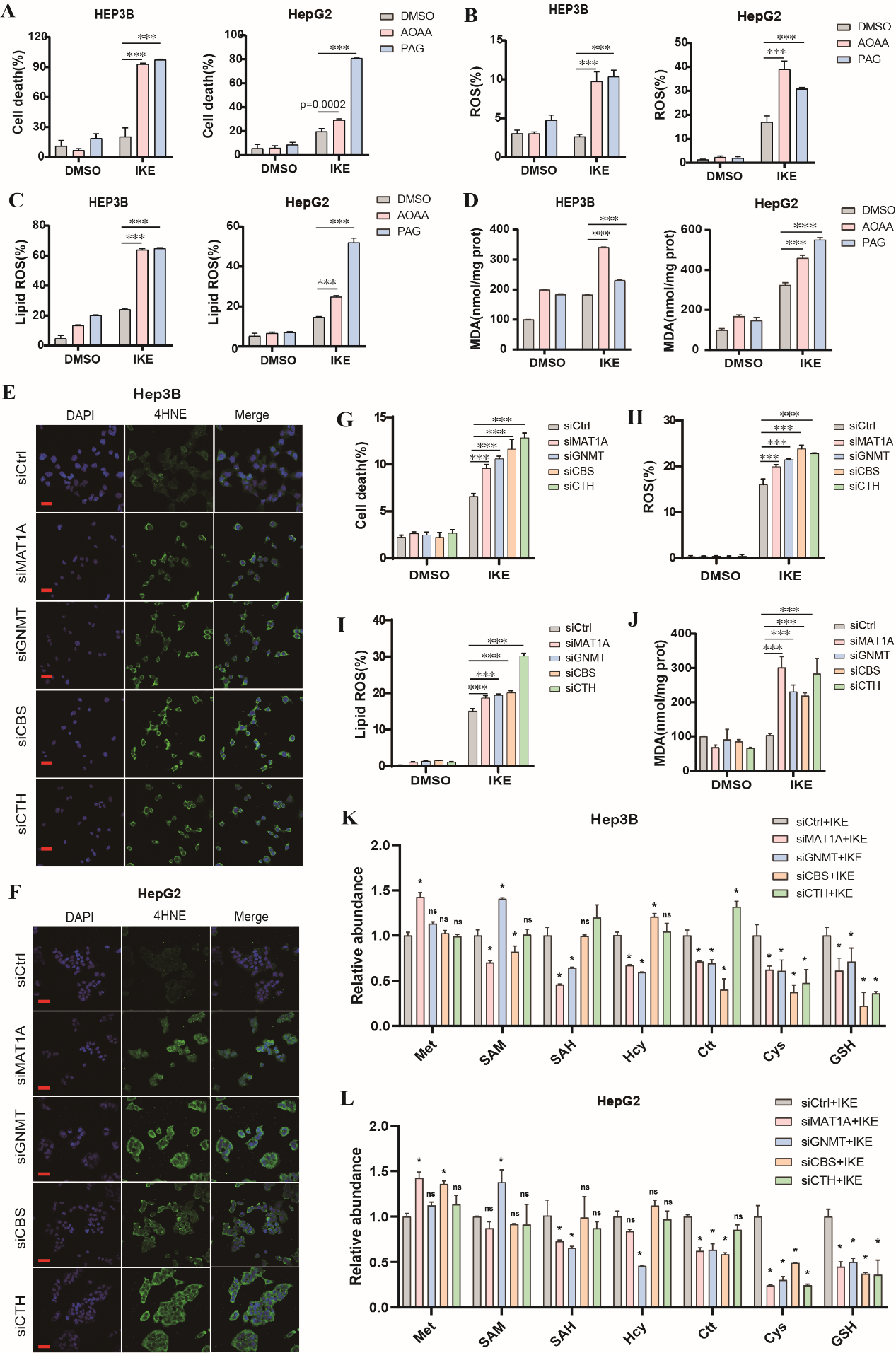


**Supplementary Fig. 2 Inhibition of methionine metabolism promoted ferroptosis in EC.**

(A-D) Hep3B and HepG2 was treated with IKE (10 µM) or DMSO combined with 5µM CBS or CTH inhibitors (AOAA, PAG) for 24 h, and cell death was assessed by 2 µM Propidium Iodide (PI) (A), ROS was assessed by 1 µM H2DCFDA (B), lipid ROS was assessed by 2 µM C11-BODIPY (581/591) (C), MDA as the final metabolite of lipid peroxidation was measured (D), ***p < 0.0001. (E-F) Cells were transfected with siRNA for 24 h, treated with IKE (10 µM) for 10 h, intracellular 4HNE (green) contents of Hep3B (E) and HepG2 (F) were measured using immunofluorescent staining, DAPI staining (blue) for nucleic acid, Scale bar = 20 µm. (G-J) After 24h of siRNA transfection, HepG2 were treated with IKE (10 µM) or DMSO for 24h, the levels of cell death(G), ROS (H), lipid ROS (I), and MDA (J) were measured. The fluorescence intensity of PI, H2DCFDA and C11-BODIPY were detected by Cytek NL-CLC full-spectrum flow cytometry (n=3), ***p < 0.001. (K-L) After 24 h of siRNA transfection, Hep3B (K) and HepG2 (L) were treated with IKE (10 µM) for 24 h, LC-MS/MS was employed to test differences of metabolites involved in methionine metabolism (n=3), ns indicates no significant difference, *p < 0.001.


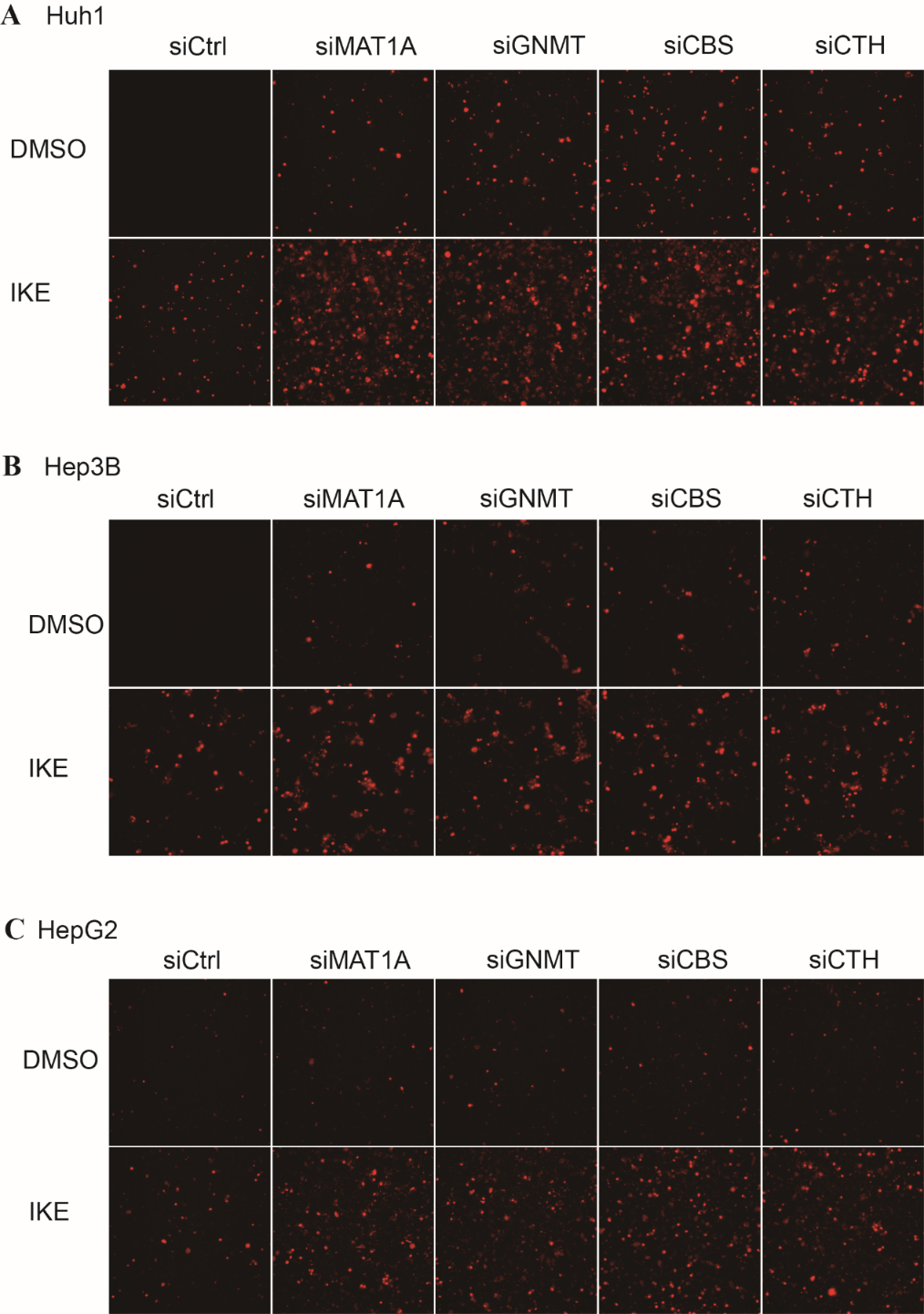


**Supplementary Fig. 3 Deficiency of major methionine metabolic enzymes increases IKE-induced cell death.**

(A-C) The sensitivity of Huh1, Hep3B and HepG2 cells to IKE was increased by knocking down the expression of MAT1A, GNMT, CBS and CTH. The Agilent eTox Red reagent (Agilent, 8711009) selectively stains the nuclei of dead cells, and the images were captured using the RTCA esight system.


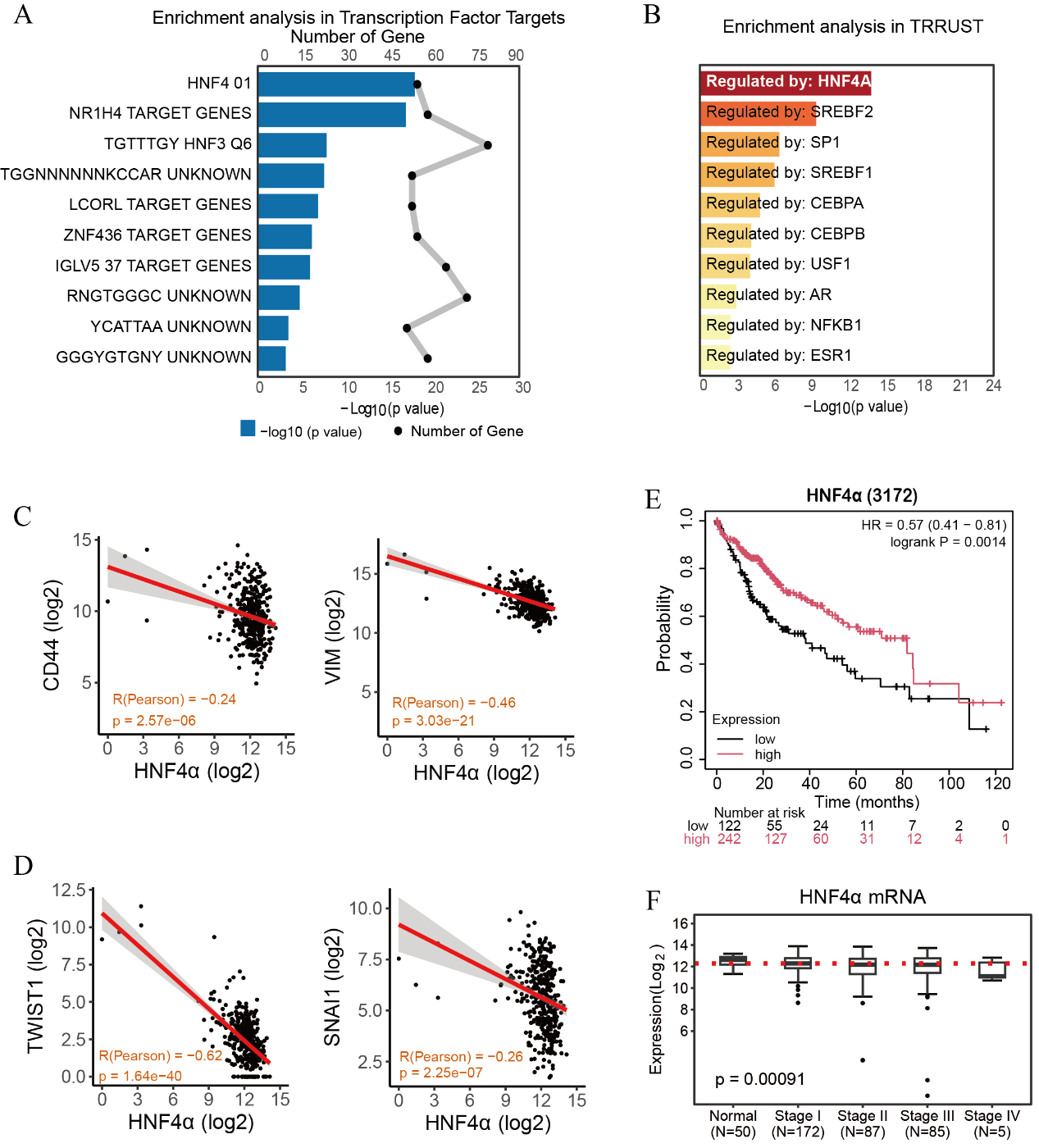


**Supplementary Fig. 4 Differentially expressed genes between EC and MC were significantly enriched in HNF4α.**

(A-B) Transcription factor target enrichment analysis and TRRUST enrichment analysis. (C-D) Scatter plot indicated that expression of mesenchymal markers (CD44 and vimentin) (C) and transcription factors regulating EMT (TWIST1 and SNAI1) (D) is negatively correlated with the expression levels of HNF4α in TCGA-LIHC patients. Pair-wise Pearson correlation coefficients and p-value between two specific genes were calculated utilizing R. (E) HNF4a high expression is associated with increased survival of HCC patients. The Kaplan-Meier survival curves were generated using KMplot website (https://kmplot.com/analysis/index. php?p=home) with liver cancer RNA-seq data and the best cutoff was automatically selected. (F) HNF4a expression is differencial in HCC patients at different stages. The TCGA RNA-seq gene expression data, processed for n=50 normal and n=373 tumor LIHC patients at different cancer stages (n=172 at Stage I, 87 at Stage II, 85 at Stage II, and 5 at Stage IV) were downloaded from TCGA data portal. Points outside the maximum and the minimum were considered as outliers.


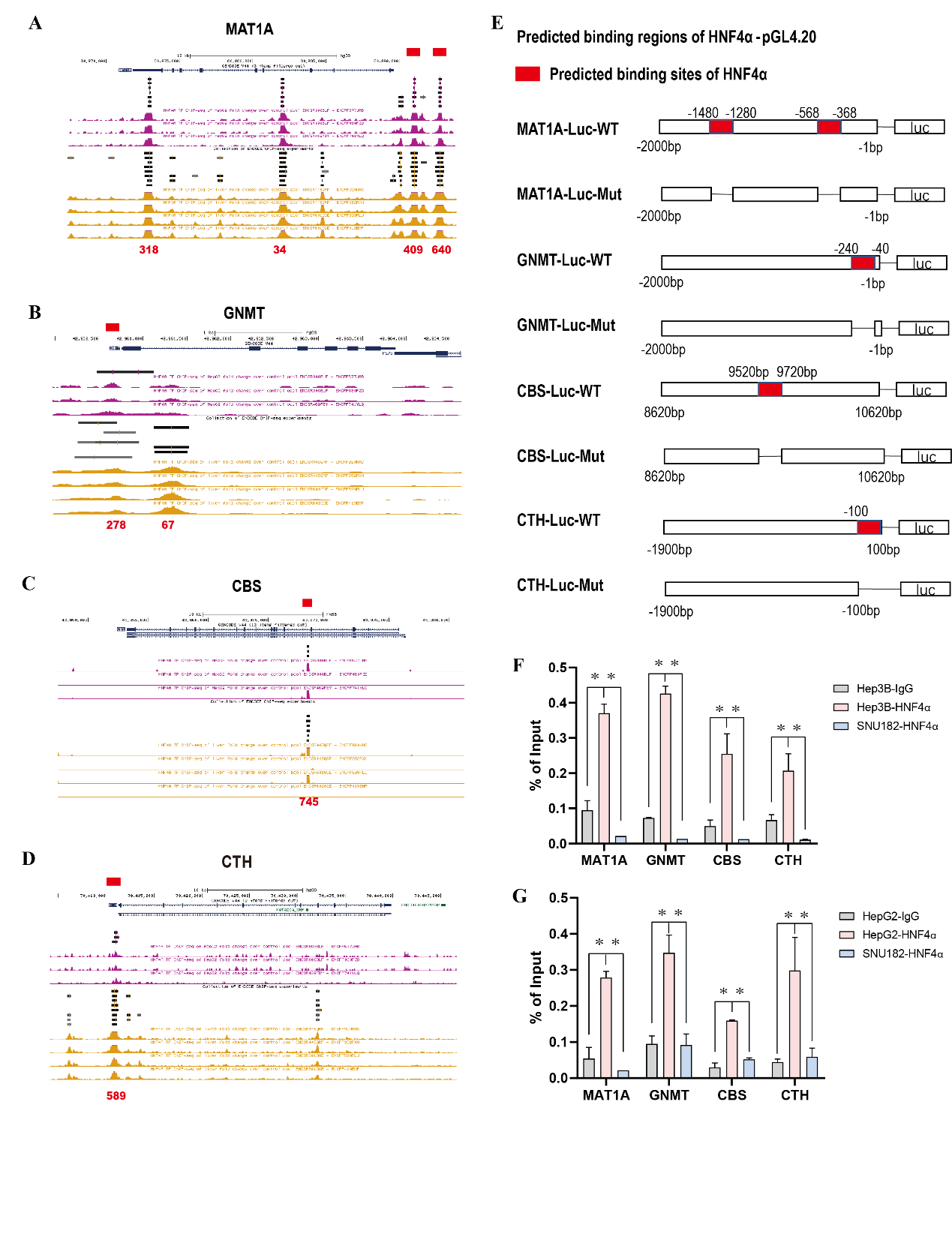


**Supplementary Fig. 5 Construction of luciferase reporter plasmids and transcriptional regulation of major** **methionine metabolic enzymes by HNF4α**

(A-D) Major methionine metabolic enzymes have HNF4a binding sites within their promoter regions. Publicly available data for the HNF4a ChIP-seq from HepG2 cells from the ENCODE project were displayed in UCSC genome browser for MAT1A (A), GNMT (B), CTH (C) and CBS (D) genes, enrichment scores were highlighted in red; Red bars: promoter regions cloned in the luciferase assay. (E) Schematic representation of the cloning of wild-type and deletion mutants of promoter fragments from MAT1A, GNMT, CTH, and CBS genes into pGL4.20-basic luciferase vector. Deletion mutant were defined as the removal of a 200-bp fragment containing the peak points. (F-G) ChIP-qPCR was performed to analyse the binding of HNF4α on the major methionine metabolic enzymes promoters in Hep3B, HepG2 and SNU182 cells. Negative controls included IgG ChIP conducted in Hep3B and HepG2 cells and anti-HNF4α ChIP conducted in SNU182 cells (n=3), *p < 0.0001.


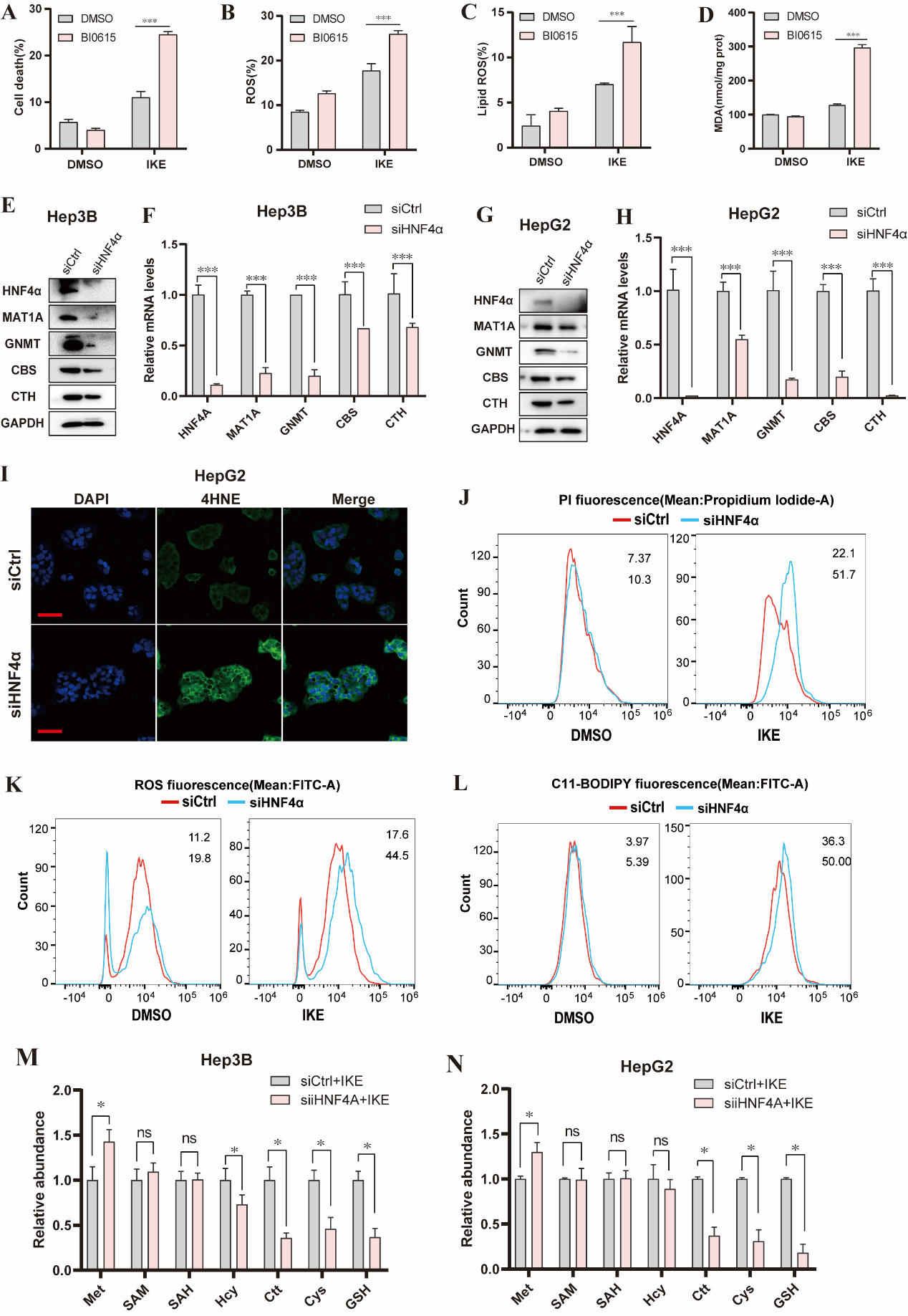


**Supplementary Fig. 6 Downregulation of HNF4α expression induces EC ferroptosis in EC.**

(A-D) HepG2 was treated with IKE (10 µM) or DMSO combined with 5µM BI6015 for 24 h, Cell death was assessed by 2 µM Propidium Iodide (PI) (A), ROS was assessed by 1 µM H2DCFDA (B), lipid ROS was assessed by 2 µM C11-BODIPY (581/591) (C), and MDA (D) were measured by lipid peroxidation (MDA) assay kit, (n=3). (E-H) After 48h of siHNF4α transfection, Western blot and RT-qPCR were performed to detect the expression of HNF4α, MAT1A, GNMT, CBS and CTH in Hep3B and HepG2 cells (n=3), ***p < 0.0001. (I) Cells were transfected with siRNA for 24 h, treated with IKE (10 µM) for 10 h, intracellular 4HNE contents were measured in HepG2 using immunofluorescent staining(green), DAPI staining (blue) for nucleic acid, Scale bar = 50 µm. (J-L) After 24h of siCtrl or siHNF4α transfection, HepG2 cells were treated with IKE (10 µM) or DMSO for 24 h, Cell death (J), ROS (K) and lipid ROS (L) were also assessed. The fluorescence intensity of PI, H2DCFDA and C11-BODIPY were detected by Cytek NL-CLC full-spectrum flow cytometry. (M-N) LC-MS/MS analysis of methionine metabolism-related metabolites in Hep3B and HepG2 cells after siRNA transfection and IKE treatment (n = 3), ns indicates no significant difference, *p < 0.001.


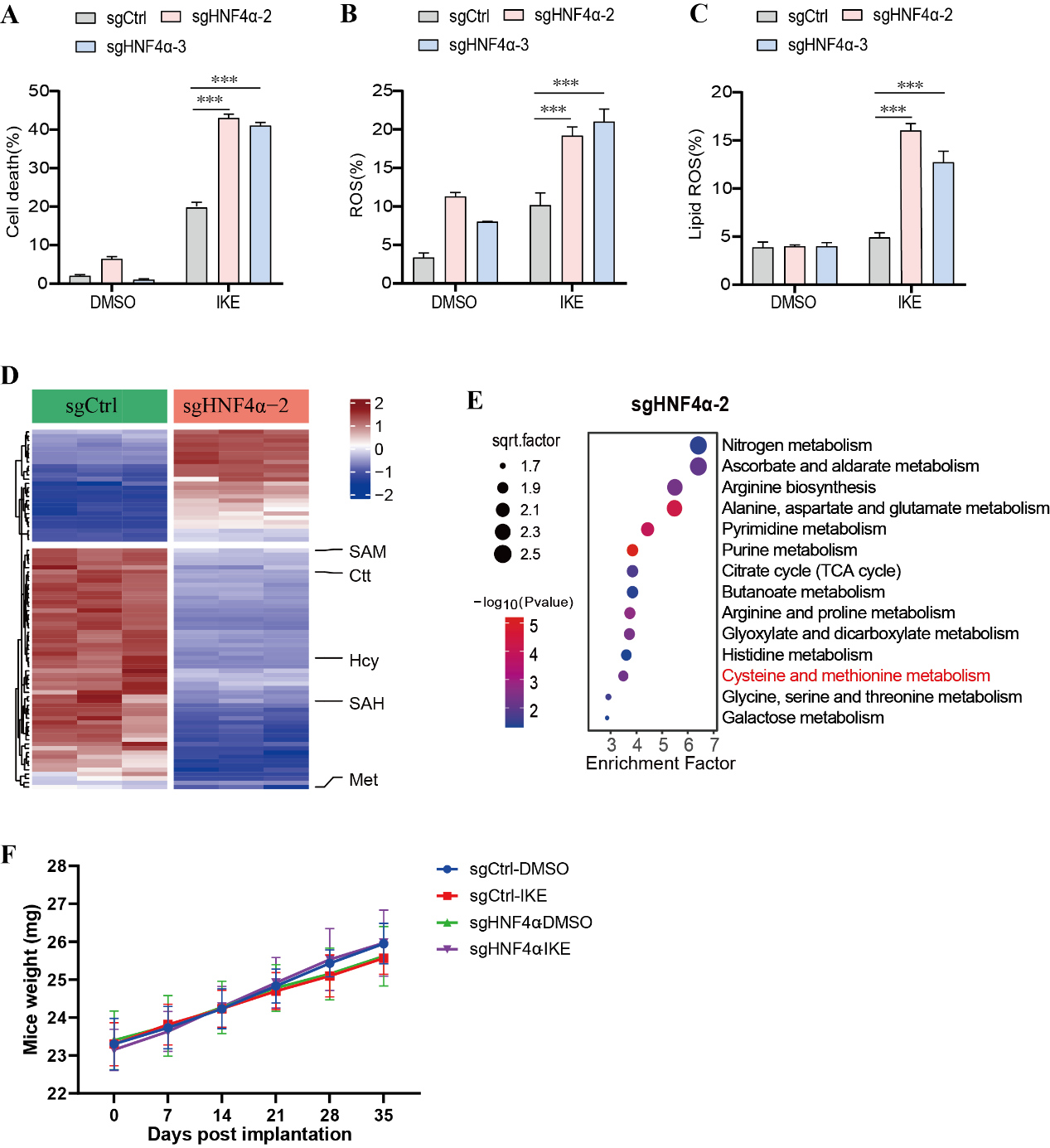


**Supplementary Fig.7 Targeting HNF4α mediates ferroptosis in vivo**

(A-C) Huh1-sgCtrl and sgHNF4α-2-3 were treated with IKE (10 µM) or DMSO, Cell death (A) was assessed by 2 µM PI, ROS (B) was assessed by 1 µM H2DCFDA, lipid ROS (C) was assessed by 2 µM C11-BODIPY (581/591). The fluorescence intensity of PI, H2DCFDA and C11-BODIPY were detected by Cytek NL-CLC full-spectrum flow cytometry (n=3), ***p < 0.0001. (D) Cluster analysis of metabolites of Huh1-sgCtrl and Huh1-sgHNF4α-2. (E) KEGG enrichment analysis was utilized on low-expression metabolites of Huh1-sgHNF4α-2. (F) Time-dependent mice weight (n=6).

**Supplementary Table 1 Sequences of primers**

| **Name** | **Sequence** |
| --- | --- |
| **qPCR primers** | |
| GAPDH-F | GTGAAGGTCGGAGTCAACGG |
| GAPDH-R | TCAATGAAGGGGTCATTGATGG |
| MAT1A-F | ATCAGGGTTTGATGTTCGGCT |
| MAT1A-R | GCGTTGAGCTTGTGAGCAA |
| GNMT-F | GCTTCAGTAAGTTTCGGCTCT |
| GNMT-R | CGATGGTCTTAGGGTGGG |
| CBS-F | TTTCGGTGCAGATGATGCAA |
| CBS-R | CATGTATCCGTCCAGGTGAGATC |
| CTH-F | TTAGCCTATTGCGTCATTTAAGCA |
| CTH-R | CTGCACCCGGTCATGAGTT |
| HNF4α-F | ACTACATCAACGACCGCCAGT |
| HNF4α-R | ATCTGCTCGATCATCTGCCAG |
| **ChIP-PCR primers** | |
| MAT1A-F | GAGAAGTTGACAGGTTAGGTG |
| MAT1A-R | CATTCTATCTTCTCAGGGAGAGC |
| GNMT-F | AGGACCTAGCCCAGGATTG |
| GNMT-R | TTAAAGCATAAGCACTGCTGGC |
| CBS-F | GGCATTGTGTCAGAGGGTGA |
| CBS-R | CACTGCTCCTTAGCAGGCAT |
| CTH-F | GTTCAGTGTACCTAAAACGC |
| CTH-R | TTACCTCTGACCACCATCC |
| **sgRNA** | |
| sgHNF4α-1 | TACGGTGCCTCGAGCTGTGA |
| sgHNF4α-2 | GCAATGACTACATTGTCCCT |
| sgHNF4α-3 | CCAAGGGGCTGAGCGATCCA |
